# Medial preoptic area in mice is capable of mediating sexually dimorphic behaviors regardless of gender

**DOI:** 10.1038/s41467-017-02648-0

**Published:** 2018-01-18

**Authors:** Yi-Chao Wei, Shao-Ran Wang, Zhuo-Lei Jiao, Wen Zhang, Jun-Kai Lin, Xing-Yu Li, Shuai-Shuai Li, Xin Zhang, Xiao-Hong Xu

**Affiliations:** 1 Institute of Neuroscience, State Key Laboratory of Neuroscience, CAS Center for Excellence in Brain Science and Intelligence Technology, Shanghai Institutes for Biological Sciences, Chinese Academy of Sciences, Shanghai, 200031 China; 2 University of the Chinese Academy of Sciences, Beijing, 100049 China

## Abstract

The medial preoptic area (mPOA) differs between males and females in nearly all species examined to date, including humans. Here, using fiber photometry recordings of Ca^2+^ transients in freely behaving mice, we show ramping activities in the mPOA that precede and correlate with sexually dimorphic display of male-typical mounting and female-typical pup retrieval. Strikingly, optogenetic stimulation of the mPOA elicits similar display of mounting and pup retrieval in both males and females. Furthermore, by means of recording, ablation, optogenetic activation, and inhibition, we show mPOA neurons expressing estrogen receptor alpha (Esr1) are essential for the sexually biased display of these behaviors. Together, these results underscore the shared layout of the brain that can mediate sex-specific behaviors in both male and female mice and provide an important functional frame to decode neural mechanisms governing sexually dimorphic behaviors in the future.

## Abstract (web summary)

The medial preoptic area (mPOA) in a mammalian brain is sexually dimorphic, and yet its exact function in mediating gender-specific behavior remains unclear. Here, Xu and colleagues show that optogenetic manipulation of the mPOA in male mice induce female-stereotyped behaviors and vice versa.

## Introduction

Mating and parental behaviors, though highly dependent on learning and experience in humans and nonhuman primates, are under strong instinctual control in many other species. Males and females of these species often behave differently during mating and toward the young as a result of sexually differentiation of the nervous system^1–3^. In mammals, gonadal secreted steroid hormones, including estrogen and testosterone, act via their receptors to specify irreversible changes in neural structures such as neuronal numbers and connections and to support sex-specific gene expression patterns and neural functions^4–8^. Yet, despite extensive characterization of sex differences in the brain at molecular and anatomical levels^9–12^ and our increased understanding of hormonally regulated genetic and epigenetic programs^8, 13–15^ that drive such sex differences, neural mechanisms governing sexually dimorphic display of behaviors remain poorly understood.

The medial preoptic area (mPOA) of the hypothalamus is a central node in a conserved neural network that regulates social behaviors and social reward^16–20^, and is anatomically sexually dimorphic in many species, including humans^21–23^. The mPOA expresses at high levels gonadal steroid hormone receptors that regulate sexually differentiation^8, 18^ and connects to other sexually dimorphic brain areas^4, 24–27^. Indeed, neuronal numbers or densities^7, 28–31^, synaptic organizations^32–34^, distributions of innervating fibers^35, 36^, and gene expression patterns^8^ in the mPOA are all found to be sexually dimorphic. Notably, sexually dimorphic features of the mPOA are sensitive to perinatal hormonal manipulations that also modify sex-specific behaviors^4, 32, 36^, therefore perhaps reflect sex-specific wirings of the underlying neural circuits that bound an animal to sex-appropriate patterns of behaviors^37^. Consistent with this notion, the mPOA is found by *c-Fos* studies to be activated during male-typical copulation and pup care^16, 20, 38^, both of which are sexually dimorphically displayed^1^. Furthermore, lesions of the mPOA disrupt these behaviors^20, 39–45^.

On the other hand, sex differences in male-typical copulation and parental care are also readily modified by hormonal, sensory, and experiential factors in adult animals^5^. For example, adult female mice treated with male-typical hormones^46^ or defect in the processing of vomeronasal information^47^ copulate females at levels comparable to intact males^46^. Likewise, male mice, which typically ignore or even attack pups as virgins, do transiently care for them as fathers^16^. These observations raise the question as how a sexually differentiated brain remains plastic for sex-atypical behaviors. Answers to this question have been largely hindered by the fact that the vast majority of functional studies often included subjects of only one sex.

In this study, we took an unbiased approach to record in vivo activities of the mPOA as well as functionally manipulated genetically defined populations of mPOA neurons during male-typical copulation and pup care in both male and female mice. Intriguingly, we find ramping activities in the mPOA that precede and correlate with sexually dimorphic display of male-typical mounting and female-typical pup retrieval, thereby establishing a direct link between mPOA neural dynamics and sexually dimorphic display of behaviors. Next, we show that optogenetic stimulation of the mPOA elicits similar display of mounting and pup retrieval in both males and females. While two recent studies^16, 17^ investigating the role of the mPOA in parental care and social reward also used optogenetic approaches to stimulate subsets of mPOA neurons, we here show that the mPOA can drive mounting and pup retrieval in an adult animal. Finally, by means of recording, ablation, optogenetic activation, and inhibition, we demonstrate that mPOA neurons expressing *Esr1* are essential for sexually biased display of these behaviors. Together, these results shed new lights on the function of the mPOA and neural organization of sexually dimorphic behaviors in general.

## Results

### mPOA neural dynamics during sexually dimorphic behaviors

**Fig. 1:**
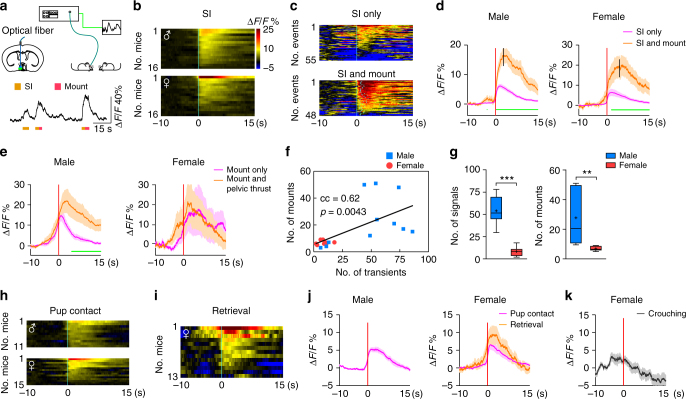
mPOA neural dynamics during social behaviors. **a** Schematics of the fiber photometry experiment. Fluorescent signals were recorded using an integrated setup. At the bottom, example traces of Δ*F*/*F* signals with behavioral annotations underneath. **b** Heat map representation of Δ*F*/*F* signals around social investigations (SI) averaged at the animal level with time “0” aligned to the onset of the behavior. Scale shown on the right applies to all heat maps in this figure. **c** Example heat map representations of Δ*F*/*F* signals around events of SI alone (top) or those that were followed by mount (bottom). Light blue lines in the bottom panel denote the onset of mount for each event. **d**, **e** Quantification of Δ*F*/*F* signals associated with SI alone or those followed by mount in males (left) and females (right) in **d**, and mount followed by pelvic thrust or not in males (left) and females (right) in **e**. Lines indicate mean values and the shaded area standard error of mean (s.e.m.). Vertical black bars in **d** denote when mount behaviors were initiated on average. Green dots underneath indicate a significant difference between the two lines at the corresponding time point by unpaired *t* test with a false discovery rate of 0.05. **d** SI, *N* = 16 males and 16 females; Mount, *N* = 13 males and 10 females; **e** *N* = 6 males and 4 females. **f** Significant correlation between total number of Ca^2+^ transients and the total number of mount between the first and last mounting behavior in a trial by Pearson’s correlation. *N* = 11 trials for males and 8 trials for females. **g** Comparison of numbers of Ca^2+^ transients (left) and numbers of mount (right) in **f** between males and females at the animal level. *N* = 8 males and 8 females. Unpaired *t* test. *p < 0.05, **p < 0.01. **h**, **i** Heat map representations of Δ*F*/*F* signals around pup contacts (**h**) and pup retrieval (**i**) at the animal level with time “0” aligned to the onset of the behavior. **j**, **k** Quantification of Δ*F*/*F* signals associated with pup contact and pup retrieval in males (left) and females (right) in **j** and crouching behavior in females in **k**

Consistent with previous studies^16^, we observed stable and quantifiable sex differences in the display of male-typical copulation and pup care in adult virgin C57BL/6 mice that correlated with sex-specific patterns of c-Fos staining in the mPOA (Supplementary Figure 1). To visualize mPOA neural dynamics during behaviors with better temporal resolution, we injected adneno-associated viruses (AAVs) encoding the fluorescent Ca^2+^ sensor GCaMP6s^48^ driven by the human synapsin promoter (hSyn) unilaterally into the mPOA of adult C57BL/6 mice and implanted optic fibers (Fig. 1a). This allows us to monitor Ca^2+^ transients as indicators of neural activities in freely behaving mice, a strategy that has proven to work in the mPOA^17^ and other brain regions^49, 50^. Control animals were injected with AAVs encoding EGFP. All recording sites were confirmed post hoc to lie within the mPOA (Supplementary Figure 2a–b).

In experimental animals of both sexes but not the controls, we observed a sharp rise in mPOA activities (average Δ*F*/*F*, 32.0 ± 2.7%) upon introduction of females or pups but not inanimate objects into the cage (Supplementary Figure 2c–f), suggesting that the mPOA preferentially processes socially relevant cues. A recent study^17^ using in vivo two-photon imaging in head fixed female mice has also identified mPOA neurons that encode social cues. After habituation of this initial spike, mPOA activities increased reliably when GCaMP6s injected animals, either male or female, physically or chemo-investigated (sniffed) a female, actions we termed collectively as “social investigations” (Fig. 1b). In complement, we found that social investigations of a female restrained under a barrier also increased mPOA activities (Supplementary Figure 2g). Importantly, investigations of an empty crib or an inanimate object did not change mPOA activities (Supplementary Figure 2g), suggesting that the observed increases in mPOA activities were unlikely motion artifacts. Intriguingly, social investigations that led to subsequent mounting were associated with larger mPOA activities than those that did not (Fig. 1c, d). Since an event of mounting was almost always preceded by an event of social investigation, mPOA activities therefore ramped before the onset of mounting in both sexes and decayed more slowly if mounts transitioned into rhythmic pelvic thrusts in males (Fig. 1e). Moreover, once mounting was initiated, the numbers of mPOA ramping events, as calculated by finding “activity bumps” that were >3 standard deviations above the baseline and lasted for >2 s, strongly correlated with the numbers of mounts, with both being more numerous in males than females (Fig. 1f, g).

Similarly, mPOA neural activities increased reliably when GCaMP6s animals, either male or female, contacted pups, and ramped prior to pup retrieval behavior, which were only displayed by female GCaMP6s animals in these experiments (Fig. 1h–j). Furthermore, the numbers of mPOA ramping events showed some correlation with the numbers of pup retrieval behavior (correlation coefficient = 0.477, *p* = 0.0752). Meanwhile, no consistent changes in mPOA activities were observed during other components of maternal care such as crouching (Fig. 1k). Together, these results show that mPOA neural activities rise following social investigations of a female and pup contacts and ramp prior to the display of male-typical copulation in both sexes and pup retrieval in females.

### mPOA activation elicits male-typical mount in both sexes

**Fig. 2:**
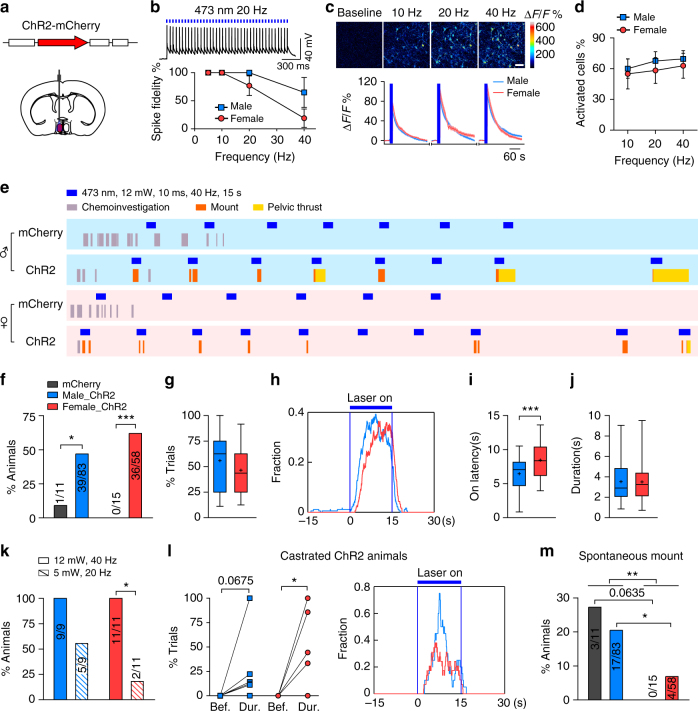
Optogenetic activation of male-typical mounting in both sexes. **a** The strategy to optogenetically activate the mPOA. **b** Electrophysiological recordings of ChR2-expressing neurons in freshly cut brain slices. *N* = 25 male cells and 23 female cells. **c** Calcium imaging of acute brain slices were performed under a two-photon microscope at different stimulation frequencies as representative images showing at the top. Δ*F*/*F* values were averaged cross cells and plotted at the bottom with the blue bars indicating laser stimulation and the line and the shading indicating the mean and the s.e.m., respectively. *N* = 358 male neurons and 292 female neurons. Scale bar, 50 μm. **d** Average percentage of neurons activated at each tested frequency. *N* = 6 brain sections for males and 4 for females. **e** Representative raster plots of behaviors (color coded) displayed by mCherry and ChR2 animals of either sex during a behavioral trial in which a hormonal primed ovariectomized female was given as the stimulus. Blue bars indicate periods of light stimulation. **f**–**j** Photostimulation elicited male-typical mounting behavior toward hormonal primed ovariectomized females. The percentage of animals that displayed mounting behavior during photostimulation (**f**), trial-by-trial occurrence (**g**), average distribution (**h**), the latency (**i**), and average duration (**j**) of optogenetically activated mounting behavior were plotted. *N* = 83 ChR2 and 11 mCherry for males, and 58 ChR2 and 15 mCherry for females. **k** Percentage of ChR2 animals that displayed light-induced mounting behavior at low power of stimulation and high power. *N* = 9 males and 11 females. **l** Occurrence and distribution of mounting behavior before (bef.), during (dur.), and after light stimulation in those ChR2 animals, which had been castrated for >3 weeks. *N* = 9 males and 6 females. **m** Spontaneous mounting behavior that occurred outside of photostimulation. *N* = 83 ChR2 and 11 mCherry for males, and 58 ChR2 and 15 mCherry for females. Fisher’s exact test in **f**, **k**, and **m**. Unpaired *t* test in **i**. Paired *t* test in **l**. *p < 0.05, **p < 0.01, ***p < 0.001

To further establish the relationship between mPOA activation and behaviors, we injected AAVs encoding channelrhodopsin 2 (ChR2)^51^ driven by the hSyn promoter unilaterally into the mPOA of adult virgin C57BL/6 mice and implanted optic fibers (Fig. 2a). Control mice were injected with AAVs encoding mCherry. We first showed by whole-cell patch clamping and by calcium imaging in acute brain slices that light pulses induced firing of action potentials in individual ChR2-expressing neurons and activation of the mPOA at a population level at different stimulation frequencies in manners that were similar between males and females (Fig. 2b–d). Post hoc analysis also confirmed that the viral infection rate was comparable between the two sexes and that the majority of ChR2-expressing neurons expressed c-Fos after light stimulation (Supplementary Figure 3a).

With this approach, we monitored animal behaviors in response to various stimuli in a neutral arena while trains of light pulses were randomly delivered. We first observed that photostimulation produced rapid locomotion that tapered gradually afterward in >90% of ChR2 animals but never in the controls (Supplementary Figure 3b–e), consistent with recent studies^16, 17^. Strikingly, when we introduced a hormonally primed female as the stimulus, a fraction of ChR2 animals (~50%), either male or female, displayed time-locked mounting behavior within seconds of photostimulation, which occasionally transitioned into rhythmic pelvic thrust (Fig. 2e, Supplementary Figure 3f–j, and Supplementary Movies 1–4). In comparison, mounting behavior displayed by control animals rarely coincided with light stimulation (Fig. 2f). The occurrence of light-induced mounting varied considerably among ChR2 animals but did not differ by the sex (Fig. 2e–g, j). Upon quantification, a subtle sex difference emerged as shown by a longer latency on average for the onset of optogenetically induced mounting in female ChR2 animals (6.5 ± 0.4 s, male vs. 8.5 ± 0.4 s, female; *p* < 0.001, Fig. 2h, i). Similarly, the fraction of animals that displayed optogenetically induced mounting dropped more steeply in female ChR2 animals with lowered stimulation power (Fig. 2k). Remarkably, light-induced mounting persisted when a male or a young rat, but not a toy mouse, was used as the stimulus or when ChR2 animals were castrated, under which conditions spontaneous mounting almost never occurred (Fig. 2l, Supplementary Figure 4a–b, and Supplementary Movies 5–7). Importantly, spontaneous mounting that happened outside of photostimulation remained sexually dimorphic and was comparable between the ChR2 and the control group in either sex (Fig. 2m). Together, these results show that optogenetic stimulation of the mPOA can bypass hormonal and sensory constraints and drive very similar display of male-typical mating in males and females, demonstrating that both sexes are equipped with functional neural circuits connected to the mPOA to execute such behaviors.

### mPOA activation elicits pup retrieval in both sexes

**Fig. 3:**
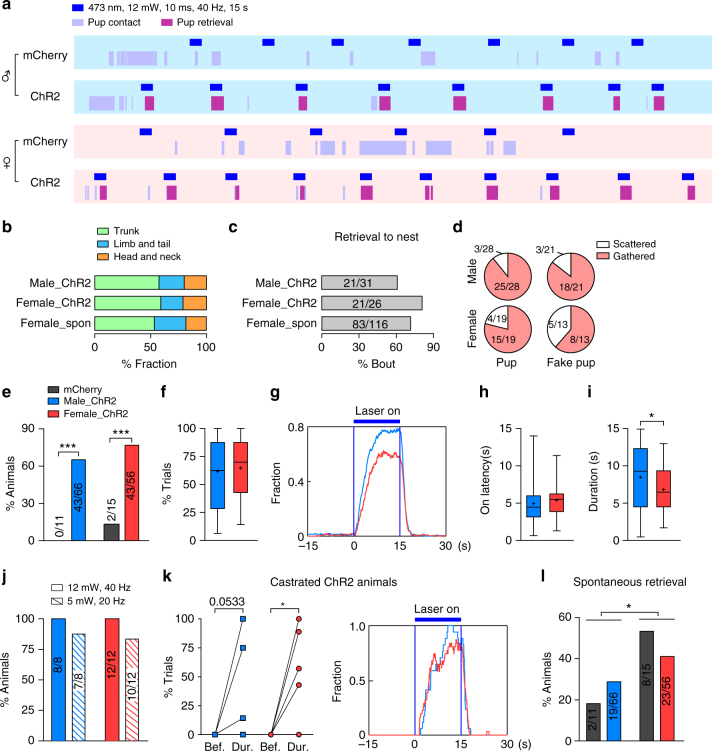
Optogenetic activation of pup retrieval in both sexes. **a** Representative raster plots of behaviors (color coded) of mCherry and ChR2 animals of either sex during a behavioral trial in which scattered pups were given as the stimuli. Blue bars indicate periods of light stimulation. **b** Body regions of a pup contacted by ChR2 animals during optogenetically induced retrieval compared with spontaneous retrieval in virgin females (female_spon). *N* = 43 ChR2 males, 42 ChR2 females, and 10 virgin females. **c** Percentage of optogenetically induced retrieval bouts that resulted in a pup being brought to the nest in male and female ChR2 animals compared to spontaneous retrieval in virgin females (female_spon). *N* = 8 ChR2 males, 5 ChR2 females, and 10 virgin females. **d** Distribution of ChR2 animals of both sexes that grouped pups or fake pups. Only animals that did not display spontaneous pup retrieval were analyzed. Grouping was defined by the appearance of ≥2 pups or fake pups together at the end of the behavioral trial. **e**–**i** Quantification of optogenetically induced pup retrieval behavior in both sexes. The percentage of animals that displayed pup retrieval behavior during photostimulation (**e**), trial-by-trial occurrence (**f**), average distribution (**g**), the latency (**h**), and average duration (**i**) of light-induced pup retrieval behaviors were plotted. *N* = 66 ChR2 and 11 mCherry for males, and 56 ChR2 and 15 mCherry for females. **j** Percentage of ChR2 animals showing light-induced retrieval behavior under low power and high power of stimulation. *N* = 8 males and 12 females. **k** Occurrence and distribution of pup retrieval before (bef.), during (dur.), and after light stimulation in ChR2 animals that had been castrated for >3 weeks. *N* = 5 males and 6 females. **l** Spontaneous pup retrieval behavior outside of photostimulation period. *N* = 66 ChR2 and 11 mCherry for males, and 56 ChR2 and 15 mCherry for females. Fisher’s exact test in **e** and **l**. Unpaired *t* test in **i**. Paired *t* test in **k**. *p < 0.05, ***p < 0.001

Concurrently, when scattered pups were given as the stimuli, a fraction of ChR2 animals (~70%), either male or female, showed time-locked pup retrieval behavior during light stimulation (Fig. 3a and Supplementary Movies 8–9). This behavior was directed specifically toward pups as beddings were never retrieved. In both sexes, pups were carried in most cases by the trunk and less frequently by head/neck or by extremities during optogenetically induced retrieval, in a manner that resembled spontaneous pup retrieval in virgin females (Fig. 3b). Moreover, if a nest was provided during the test, ~70% bouts of optogenetically induced retrieval resulted in a pup being brought to the nest, a rate that was also comparable to spontaneous pup retrieval by virgin females (Fig. 3c and Supplementary Movie 8). In the absence of a nest, pups tended to be grouped together after a series of optogenetically induced retrieval (Fig. 3d and Supplementary Movie 9). Interestingly, fake pups made of rubber blocks when given as the stimuli were also retrieved or grouped during mPOA optogenetic stimulation (Fig. 3d, Supplementary Figure 4c, and Supplementary Movie 10). The occurrence and onset latency of optogenetically induced pup retrieval were similar between the two sexes (Fig. 3e–h) while the average amount of time spent carrying pups during a photostimulation period was slightly longer in ChR2 males than females (8.5 ± 0.6 s, male vs. 6.8 ± 0.4 s, female; *p* < 0.05; Fig. 3i). Furthermore, in both sexes optogenetically induced retrieval persisted under lowered power of stimulation or after castration (Fig. 3j–k). By contrast, spontaneous pup retrieval behavior outside of photostimulation remained female biased and was comparable between ChR2 and control animals (Fig. 3l). Together, these results show that optogenetic stimulation of the mPOA elicits similar pup retrieval behavior in both males and females despite female-biased display of such a behavior normally, suggesting the existence of mPOA-connected neural substrates supporting pup retrieval in both sexes, which perhaps underlie facilitation or induction of this behavior by hormonal and experiential factors in virgin or pregnant females and in males^52^.

### mPOA activation correlates with induced behaviors

**Fig. 4:**
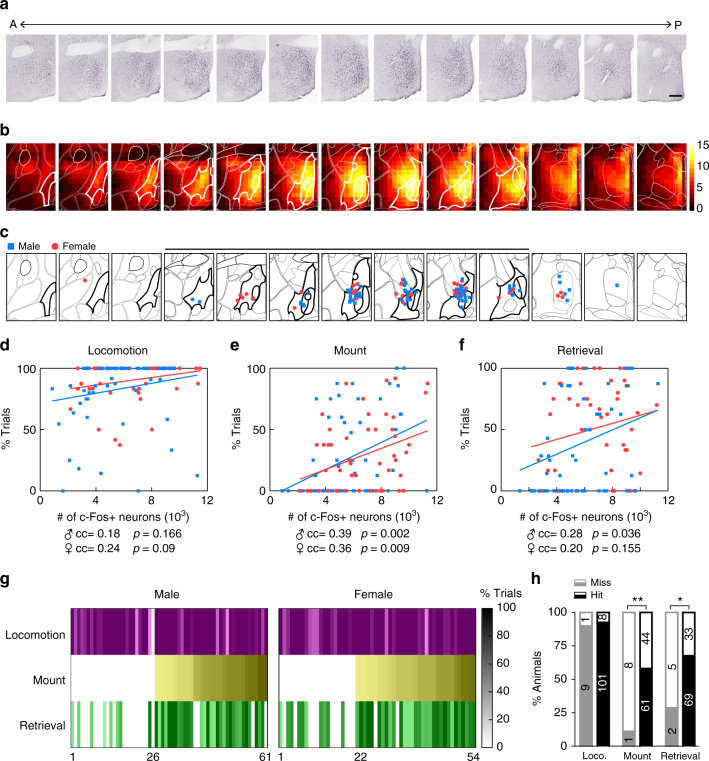
Correlation between optogenetically induced c-Fos expression and behaviors. **a** Example images of DAB staining of light-induced c-Fos expression in a ChR2 animal along the anterior–posterior axis at ~80 μm intervals. Scale bar, 200 μm. **b** Heat map representation of light-induced c-Fos expression averaged across animals. Briefly, c-Fos signals within each 100 μm^2^ square in a 1100 × 1500 μm area as in **a** were counted, tallied, averaged, plotted, and projected onto a reference atlas (http://www.brain-map.org/, image 49–61). White bold lines highlight the mPOA defined by the atlas. Scale shown on the right. **c** Activation centers for ChR2 animals. *N* = 66 males and 53 females. **d**–**f** Correlation between total number of c-Fos signals in sections highlighted with the thick black line in **c** and the trial occurrence of light-induced locomotion (**d**), mount (**e**), and pup retrieval (**f**) for both male and female ChR2 animals. Pearson’s correlation. **g** Distribution of optogenetic-induced locomotion, mount, and pup retrieval (color coded) in ChR2 males (left) and females (right) with each column corresponding to a ChR2 animal and the shade of each cell corresponding to the trial occurrence of the behavior indicated on the left. The scale bar is on the right. Animals were sorted according to the occurrence of light-induced mounting behavior. Out of 35 males and 33 females that displayed light-induced mounting, 31 males and 31 females also displayed light-induced pup retrieval. Of 26 males and 22 females that did not display light-induced mounting, only 8 males and 10 females displayed light-induced pup retrieval. **h** Categorical comparison of the occurrence of light-induced locomotion, mount, and pup retrieval at the animal level between groups whose activation center lie within or close to the mPOA, as in sections highlighted with a black line in **c** (hit), and those away from the mPOA (miss). Filled segments indicate the fraction of animals in each group that displayed optogenetically induced behaviors listed below while corresponding empty segments indicate the fraction that did not display the behavior. Fisher’s exact test. *p < 0.05, **p = 0.01

All animals tested for behaviors were analyzed post hoc to verify viral infection with the majority also stained for light-induced c-Fos expression and measured for serum hormone levels. These analysis confirmed that mPOA was consistently activated across ChR2 animals (Fig. 4a, b) and that optogenetic activation did not alter serum testosterone levels (male, 11 control vs. 41 ChR2: 0.46 ± 0.087 vs. 1.38 ± 0.34 ng ml^−1^, *p* = 0.17; female, 15 control vs. 25 ChR2: 0.14 ± 0.0073 vs. 0.13 ± 0.012 ng ml^−1^, *p* = 0.53), which remained higher in males in both ChR2 and control groups (two-way ANOVA, *p* < 0.001). Moreover, by calculating the site that had the densest c-Fos staining along the anterior–posterior, dorsal–ventral, and medial–lateral axes, we simulated an activation center for each ChR2 animal (see “Methods” section) and showed that they predominantly clustered within or close to the mPOA as defined by the atlas (Fig. 4c, http://www.brain-map.org/). In fact, the total number of neurons activated in the mPOA and adjacent areas, as measured by the sum-of-light-induced c-Fos signals in these regions, correlated with light-induced behaviors (Fig. 4d, f). Consistently, optogenetically induced behaviors co-occurred at the animal level (Fig. 4g). In a subset of animals that displayed both optogenetically induced mounting and pup retrieval, we stimulated the mPOA at two different power in the presence of both a female and scattered pups. We found that these animals mounted the female or retrieved pups at random during photostimulation with no apparent bias in either sex (Supplementary Table 1). Occasionally, they even displayed both behaviors in tandem within a single light stimulation (Supplementary Movie 11).

Extensive efforts of varying injection sites and viral amounts failed to further segregate optogenetic-induced behaviors, indicating that neurons responsible for these behaviors likely intermingle closely and may even overlap. Nevertheless, animals whose activation center lied away from the mPOA were less likely to display optogenetically induced mounting or pup retrieval (Fig. 4h), supporting the notion that stimulation of the mPOA drives the display of male-typical mounting and female-typical pup retrieval in both sexes.

### mPOA *Esr1*+ neurons activated during mount and pup retrieval

Given that pan-neuronal activation of the mPOA elicited similar behaviors in both males and females, to explore possible neural mechanisms underlying sexually dimorphic display of mounting and pup retrieval, we targeted a subset of mPOA neurons that express estrogen receptor α (*ERα* or *Esr1*), a gene that regulates sexual differentiation and reproductive behaviors^4^. Esr1+ cells constitute ~50% of mPOA neurons (male, *N* = 3, 53 ± 5.5%; female, *N* = 3, 53 ± 3.3%) and overlap with both *Vgat* and *Vglut2*, with *Vgat*+ neurons making up ~80% of all NeuN+/HuCD+ cells in this region (male, *N* = 3, 78 ± 3.0%; female, *N* = 3, 76 ± 4.9%) (Supplementary Figure 5a–b). In addition, a fraction of Esr1+ neurons (15–30%), higher in males, express the neuropeptide *Galanin* and account for about ~50% of the mPOA *Galanin*+ neurons (Supplementary Figure 5c), which has been shown previously to regulate parental care^16^.

**Fig. 5:**
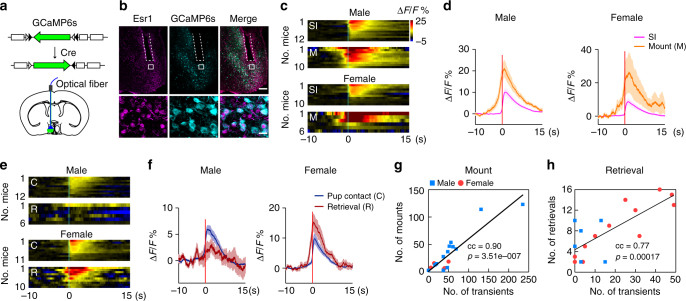
Neural dynamics of mPOA *Esr1*+ neurons during social behaviors. **a** Schematics of the strategy to record activities of *Esr1*+ neurons. **b** Example images showing co-localization of GCaMP6s and Esr1 signals. Images on the bottom are blowups of the corresponding white squares in the images on the top. Dashed lines show the placement of the optic fiber. Scale bar represents 200 μm for images on the top and 20 μm for images at the bottom. **c**–**f** Heat map plots (**c**, **e**) and quantification (**d**, **f**) of Δ*F*/*F* signals around events of social investigations (SI) and mount (M) in male and female Esr1^Cre^ GCaMP6s animals during social interactions with a female or around events of pup contacts (C) and pup retrieval (R) during interactions with scattered pups, with time “0” aligned to the onset of behaviors. Scale bar in **c** applies to all heat maps in this figure. In **d** and **f**, lines indicate the mean and the shaded area the s.e.m. *N* = 12 males and 11 females. **g**, **h** Significant correlation between total number of Ca^2+^ transients in mPOA *Esr1*+ neurons and the total number of mount (**g**) and retrieval (**h**) between the first and last behavior in a trial. Mount, *N* = 13 trials for males and 5 trials for females; retrieval, *N* = 6 trials for males and 12 trials for females. Pearson’s correlation

Utilizing a knock-in Cre line (Esr1-2A-Cre, Esr1^Cre^) generated previously^53^, we first validated the fidelity of *Cre* expression and then injected AAVs encoding Cre-inducible Gcamp6s into the mPOA of Esr1^Cre^ animals to record activities of *Esr1*+ neurons (Fig. 5a, b, Supplementary Figure 5d, and Supplementary Figure 6a). Similar to mPOA pan-neuronal recordings, activities of *Esr1*+ neurons increased reliably following social investigations of a female and pup contacts and ramped prior to the onset of mounting in both males and females and pup retrieval in females (Fig. 5c–f). Additionally, events of ramping activities in mPOA *Esr1*+ neurons strongly correlated with events of mounting, more prevalent in males, and pup retrieval, more prevalent in female (Fig. 5g, h). In comparison, when we injected Cre-silenced Gcamp6s to monitor activities of mPOA *non-Esr1*+ neurons, much less signals were detected during any phase of social interactions with a female or pups (Supplementary Figure 6b–f, h–i), despite robust activation observed in control wild-type animals injected with the same virus, which now labeled both *Esr1*+ and *non-Esr1*+ neurons (Supplementary Figure 6g, j).

**Fig. 6:**
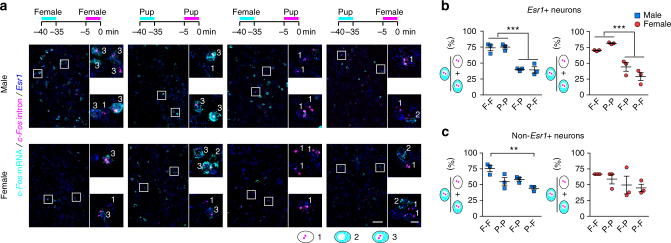
catFISH analysis of mPOA neuronal activation during social interactions with a female or scattered pups. **a** Following two sequential social interactions with either a female (F) or scattered pups (P), as indicated at the top, animals were processed to stain for *c-Fos* mRNA, shown in cyan, *c-Fos* intron in magenta, and *Esr1* mRNA in blue with DAPI as the counter staining (not shown). Images on the right are blown ups of white boxes in images on the left with numbers indicating whether the identified nearby cell expresses nuclear *c-Fos* only (1), or cytoplasmic *c-Fos* only (2), or both (3). Scale bar represents 50 μm for images on the left and 10 μm for images on the right. **b**, **c** Quantification of the percentage of *Esr1*+ neurons (**b**) and *non-Esr1*+ neurons (**c**) with nuclear *c-Fos* that co-expressed cytoplasmic *c-Fos* under each test condition. F-F female-female, P-P pup-pup, F-P female-pup, P-F pup-female. For Esr1+ neurons, the percentage was significantly higher in animals exposed to the same stimuli compared to those to different stimuli. For *non-Esr1*+ neurons, the percentage was only found to be different between the “F-F” and “P-F” group in males. One-way ANOVA with Bonferroni correction. *N* = 3 per condition per sex. **p < 0.01, ***p < 0.001

In addition, using the method of cellular compartmental analysis of temporal activity by fluorescent in situ hybridization (catFISH)^16, 38^, we compared populations of mPOA *Esr1*+ neurons that were activated in response to a female or scattered pups in the same animal. Briefly, animals were allowed two 5-min episodes of social interactions with either a female or scattered pups, separated apart by 30 min, and processed immediately afterward to examine the expression of nuclear and cytoplasmic *c-Fos* transcripts along with *Esr1* (Fig. 6a). Approximately 50% of *c-Fos*+ cells were *Esr1*+. By analyzing the percentage of *Esr1*+ neurons that expressed *c-Fos* transcripts, either nuclear or cytoplasmic, it was first estimated that ~10–20% *Esr1*+ neurons were activated by either stimulus. Notably, in animals exposed to the same stimulus twice, ~70–80% of mPOA *Esr1*+ neurons with nuclear *c-Fos* also stained positive for cytoplasmic *c-Fos*, indicating activation of similar neural ensemble (Fig. 6a, b). By contrast, in animals exposed to two different stimuli, only ~30–40% of co-localization was observed (Fig. 6b), which though higher than expected by chance (10–20%) was much lower than the co-localization rate observed in animals presented with the same stimulus, indicating that mPOA *Esr1*+ neurons activated during social interactions with a female or scattered pups were largely distinct. Interestingly, such a separation was not apparent in *non-Esr1*+ neurons (Fig. 6c). Taken together, these results show activation of distinct mPOA *Esr1*+ neurons during social interactions with a female or pups.

### mPOA *Esr1+* neurons key for sexually dimorphic behaviors

**Fig. 7:**
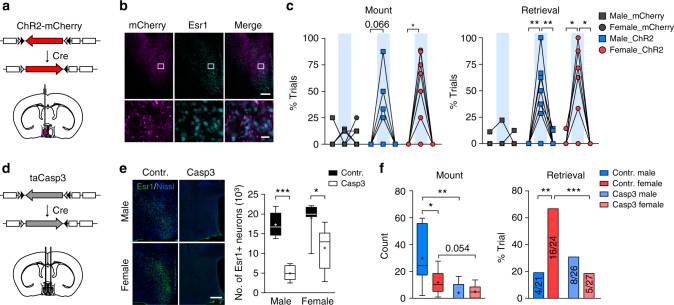
Effects of activating and ablating mPOA *Esr1+* neurons on mount and pup retrieval. **a**–**c** Optogenetic activation of mPOA *Esr1*+ neurons elicited mount and pup retrieval. **a** AAVs encoding Cre-inducible ChR2 were injected unilaterally into the mPOA of Esr1^Cre^ animals. **b** Representative images showing expression of ChR2 as indicated by mCherry signal in Esr1+ neurons. Scale bar represents 200 μm for images on the top and 20 μm for images at the bottom. **c** Percentage of trials that animals displayed mount or pup retrieval in mCherry and ChR2 animals before, during (blue bars), and after phasic photostimulation (12 mW, 10 ms, 40 Hz, 15 s). For mount, *N* = 8 ChR2 and 9 mCherry males and 10 ChR2 and 8 mCherry females. For retrieval, *N* = 9 ChR2 and 9 mCherry males and 10 ChR2 and 8 mCherry females. Paired *t* test. *p < 0.05, **p < 0.01. **d**–**f** Ablation of mPOA *Esr1*+ neurons abolished sex differences in mount and pup retrieval. **d** AAVs encoding Cre-inducible taCasp3 were injected bilaterally into the mPOA of Esr1^Cre^ animals to ablate *Esr1*+ neurons. **e** Example images of Esr1+ immunostaining in green and Nissl as the counter staining in blue in control and experimental animals of both sexes with quantification of Esr1+ signals shown on the right. Two-way ANOVA followed by Bonferroni post tests showed: virus effect, *F*(1, 22) = 44.43, *p* < 0.0001, sex effect, *F*(1, 22) = 7.38, *p* < 0.05, interaction effect, *F*(1, 22) = 2.52, *p* = 0.127. *N* = 8 Casp3 and 5 control males and 6 Casp3 and 7 control females. Scale bar, 300 μm. **f** Sex differences in mounting behavior and pup retrieval were abolished in Esr1^Cre^ animals injected with Casp3 virus. Mount, two-way ANOVA followed by Bonferroni post tests for mount count: virus effect, *F*(1, 29) = 17.28, *p* < 0.001, sex effect, *F*(1,29) = 4.79, *p* < 0.05, interaction effect, *F*(1, 29) = 5.74, *p* < 0.05. Retrieval, two tailed Fisher’s exact test, *p* < 0.001, Casp3 vs. control in females. *N* = 9 Casp3 and 7 control males and 9 Casp3 and 8 control females

To prove functionally that mPOA *Esr1*+ neurons regulate mounting and pup retrieval, we injected AAVs encoding Cre-dependent ChR2 or control virus unilaterally into the mPOA of Esr1^Cre^ animals (Fig. 7a, b). Indeed, optogenetic stimulation of mPOA *Esr1*+ neurons elicited similar display of male-typical mounting and pup retrieval in both males and females with an efficacy that was comparable to pan-neuronal activation (Fig. 7c). Next, we injected AAVs encoding Cre-dependent taCasp3^54^ bilaterally into Esr1^Cre^ animals to ablate mPOA *Esr1*+ neurons (Fig. 7d). Control animals included Cre mice injected with AAVs encoding mCherry and wild-type littermates injected with the taCasp3 virus. Successful targeting was confirmed by post hoc staining showing reduction of Esr1+ signals in experimental animals compared to the controls (Fig. 7e). Strikingly, ablation of *Esr1*+ neurons essentially abolished sex-biased display of mounting and pup retrieval by more severely impairing mounting in males and pup retrieval in females (Fig. 7f). Importantly, locomotion, chemoinvestigation, pup contacts, and sexually dimorphic display of territorial aggression were not affected after ablation of mPOA *Esr1*+ neurons (Supplementary Figure 7). In comparison, ablation of mPOA *Vgat* + or *Vglut2* + neurons, though also greatly reduced the occurrence of male-typical mounting and pup retrieval, failed to affect the sexually dimorphic nature of these behaviors such that pup retrieval remained female biased after ablation of *Vgat*+ neurons and mounting remained male biased after ablation of *Vglut2*+ neurons (Supplementary Figure 8). With the caveats of chronic and non-specific effects inherent to lesion studies^55^ (Supplementary Figure 9), these functional studies strongly implicate mPOA *Esr1*+ neurons as the key population that underlie sexually dimorphic display of mounting and pup retrieval.

### Acute inhibition of mPOA *Esr1*+ neurons disrupts behaviors

**Fig. 8:**
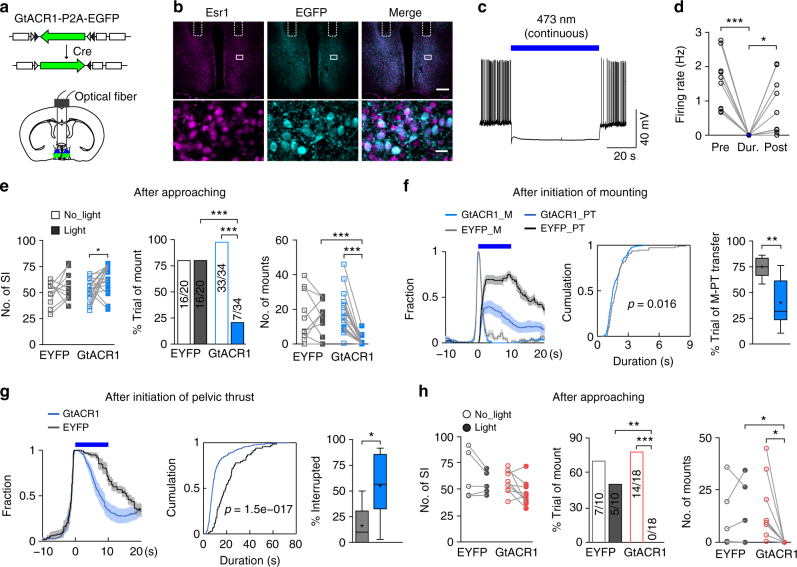
Optogenetic inhibition of mPOA *Esr1*+ neurons disrupts male-typical mating in both sexes. **a** Schematics of the strategy to optogenetically inhibit mPOA *Esr1*+ neurons. **b** Representative images showing expression of GtACR1 as indicated by EGFP in Esr1+ neurons. Scale bar, 300 μm, top; 20 μm, bottom. **c** Electrophysiological recordings of GtACR1-expressing neurons. Action potentials induced by withholding the membrane potential −48 mW with a current injection of 25 pA were blocked under continuous blue light stimulation. **d** Quantification of firing rates before (pre), during (dur.), and after (post) light stimulation. *N* = 5 male cells and 5 female cells. Paired *t* test. **e** Light delivery was triggered when the tested animal was within one body length distance to the female stimulus. Number of social investigation, percentage of trials with mount behavior and number of mounts for GtACR1 and control males in light and no-light trials were plotted and compared. *N* = 10 EGFP and 17 GtACR1 males. **f** A 10 s light pulse was delivered after the initiation of mount. The distribution of mount (M) and pelvic thrust (PT), the cumulative distribution of mount duration and the percentage trials with mount transitioning into pelvic thrust were plotted and compared between EYFP and GtACR1 males. *N* = 7 EYFP and 9 GtACR1 males. **g** A 10 s light pulse was delivered after the initiation of pelvic thrust. The distribution of pelvic thrust (PT), the cumulative distribution of duration of pelvic thrust and the percentage trials with pelvic thrust terminating during light stimulation were plotted and compared between EYPF and GtACR1 males. *N* = 7 EYFP and 9 GtACR1 males. **h** Females were treated with subcutaneous injection of testosterone. Light delivery was triggered when the tested animal was within one body length distance to the female stimulus. Number of social investigation, percentage of trials with mount behavior, and number of mounts in GtACR1 and control females during light or no-light trials were plotted and compared. *N* = 5 EYFP and 9 GtACR1 females. In **e** and **h**, *t* test or Wilcoxon rank-sum test, left and right, Fisher’s exact test, middle. In **f** and **g**, two-sample ks-test, middle, unpaired *t* test, right. *p < 0.05, **p < 0.01, ***p < 0.001

Finally, to test whether activities of mPOA *Esr1*+ neurons acutely regulate mounting or pup retrieval, we expressed the *Guillardia theta* anion channelrhodopsin 1 (GtACR1)^56^ into *Esr1*+ neurons by injecting AAVs encoding Cre-inducible GtACR1 bilaterally into the mPOA of Esr1^Cre^ animals and implanted optic fibers (Fig. 8a, b). We confirmed by whole-cell patch clamp recording in brain slices that GtACR1-expressing *Esr1*+ neurons were effectively silenced by repeated delivery of continuous blue light with no detectable rebound activities or cytotoxicity (Fig. 8c, d). Control Esr1^Cre^ animals were injected with AAVs encoding Cre-inducible EYFP. Blue light was delivered either following appetitive approaching behavior or after the initiation of consummatory acts to assess the role of activities in *Esr1*+ neurons at different phases of social interactions.

During tests of mating behaviors, light stimulation when the tested animals were within one body length distance to a female greatly reduced the percentage of trials that mounting occurred and the numbers of mounts, without decreasing social investigation, in GtACR1 males but not the controls (Fig. 8e). By comparison, delivery of a 10 s pulse of light right after the initiation of mounting only slightly shifted mount duration to be shorter in GtACR1 males but dramatically decreased the fraction of mounts that transitioned into rhythmic pelvic thrusts (Fig. 8f). Furthermore, a 10 s pulse light after the initiation of a rhythmic pelvic thrust drastically decreased the duration of the behavior, such that it more likely terminated during the stimulation period in GtACR1 males compared to the controls (Fig. 8g). In parallel, blocking activities of mPOA *Esr1*+ neurons also appeared to reduce male-typical mounting in GtACR1 females but the baseline levels were too low to permit meaningful statistical analysis. To circumvent this, we treated females with subcutaneous injection of testosterone to augment male-typical mounting^46^ and found that light delivery following approaching essentially eliminated male-typical mounting without affecting social investigation in testosterone-treated GtACR1 females but not the controls (Fig. 8h). Taken together, these results demonstrate that activities of mPOA *Esr1+* neurons are required for male-typical mounting behavior in both males and females.

**Fig. 9:**
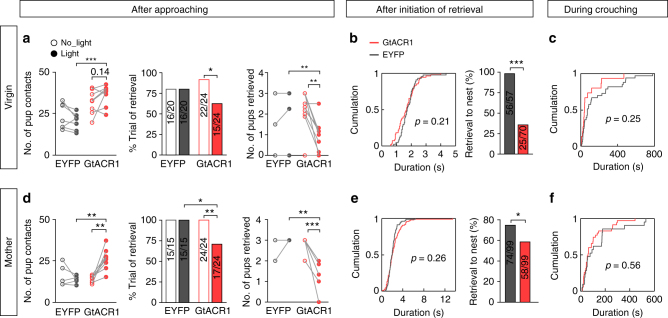
Optogenetic inhibition of mPOA *Esr1*+ neurons disrupts pup retrieval in both virgin and lactating females. **a** In virgin females, light was delivered when animals were within pre-specified distance to pups, number of pup contacts, percentage of trials with pup retrieval behavior, and number of pups retrieved to the nest were plotted and compared between no-light and light trials for both GtACR1 and control females. *N* = 7 EYFP and 8 GtACR1. **b** In virgin females, a 5 s light pulse was delivered after initiation of each retrieval, cumulative distribution of retrieval duration and the percentage of retrieval bouts that brought a pup to the nest were plotted and compared between GtACR1 and control females. *N* = 3 EYFP and 3 GtACR1. **c** In virgin females, 10 s light pulses were delivered during crouching, cumulative distribution of crouching duration was plotted and compared between GtACR1 and control females. *N* = 3 EYFP and 2 GtACR1. **d**–**f** In lactating females, light was delivered when animals were within pre-specified distance to pups (**d**), or a 5 s light pulse was delivered after initiation of retrieval (**e**), or 10 s light pulses were delivered during crouching (**f**), and similar parameters were plotted as in virgin females. In **d**, *N* = 5 EYFP and 8 GtACR1; in **e**, *N* = 3 EYFP and 6 GtACR1; In **f**, *N* = 3 EYFP and 5 GtACR1. In **a** and **d**, *t* test or Wilcoxon rank sum test, left and right, Fisher’s exact test, middle. In **b** and **e**, ks-test, left, Fisher’s exact test, right. *p < 0.05, **p < 0.01, ***p < 0.001

Similarly, during test of maternal behaviors, light stimulation in virgin females when the tested animals were within a pre-specified distance to pups significantly decreased the percentage of trials with retrieval behavior and the numbers of pups that were retrieved to the nest, without affecting pup contacts, in GtACR1 animals but not the controls (Fig. 9a). The same stimulation protocol in GtACR1 fathers also appeared to inhibit pup retrieval (*N* = 2). In complement, a 5 s pulse light stimulation after the initiation of pup retrieval did not terminate or decrease the duration of retrieval in the GtACR1 females but resulted in a dramatic reduction in the rate that a pup was retrieved to the nest (Fig. 9b). By contrast, photoinhibition during crouching had no obvious effects on the behavior (Fig. 9c). Furthermore, we mated females to deliver pups and tested the effects of photoinhibiting mPOA *Esr1*+ neurons on maternal behaviors in lactating females. With some minor differences on the effect size, essentially all behavioral deficits observed in virgin GtACR1 females with photoinhibition were also found in lactating females (Fig. 9d–f). Thus, activities in mPOA *Esr1*+ neurons regulate pup retrieval in both virgin and lactating females and in fathers. Taken together, our data support a model that sexually dimorphic activation of mPOA *Esr1*+ neurons underlie sex-biased display of mounting and pup retrieval.

## Discussion

Despite extensive characterization of sex differences in the brain at the molecular and anatomical levels, neural organization of sexually dimorphic behaviors remains unclear. Here, by recording in vivo activities of mPOA neurons during male-typical mating and pup care as well as functional manipulating genetically defined neuronal populations, we find that the mPOA in a fully sexually differentiated adult animal maintains the capacity to drive either male-typical mounting and female-typical pup retrieval and that sexually dimorphic engagements of mPOA *Esr1*+ neurons likely underlie the observed sex differences in these behaviors. Our results serve as a foundation for future investigation on neural mechanisms governing sexually dimorphic display of behaviors.

By applying fiber photometry along with viral expression of a genetically encoded calcium indicator, we found that activities of the mPOA, in particular *Esr1*+ neurons, increased following social investigations of a female or pup contacts, behaviors that are generally considered as appetitive, and ramped preceding consummatory acts of male-typical mounting or pup retrieval. While previous c-Fos studies^16, 20^ have demonstrated collective activation of the mPOA during male-typical mating and parental care, such studies lacked the necessary temporal resolution to separate out mPOA neural dynamics during different behavioral components. Indeed, results from our in vivo recording experiments, immediately suggest that the mPOA regulates consummatory actions. Supporting this, ablating mPOA *Esr1*+ neurons or inhibiting their activities dramatically disrupted male-typical mating and pup retrieval without decreasing appetitive social interactions, while activation of the mPOA or mPOA *Esr1*+ neurons drove male-typical mating and pup retrieval in both sexes. These results are especially interesting when considered in juxtaposition with a previous study^53^, which has assigned a more central role for *Esr1*+ neurons in the ventrolateral division of the ventromedial hypothalamus (VMHvl) in controlling social investigations toward females. VMHvl reciprocally connects with the mPOA. Activation of VMHvl *Esr1*+ neurons was both necessary and sufficient for close investigations toward a female, and was sufficient but not necessary for male-typical mounting. In fact, VMHvl neural activities declined during consummatory phase of male mating^38^. Furthermore, unlike the mPOA, acute inhibition of the VMHvl or VMHvl *Esr1*+ neurons could not disrupt male-typical mating^38, 53^. Thus, it seems that the mPOA is more important for consummatory acts of mating and pup retrieval while VMHvl, perhaps along with other inputs to the mPOA, controls appetitive social interactions and activation of the mPOA during such phases.

It is rather striking that optogenetic stimulation of the mPOA elicited the display of mounting and pup retrieval that were to the large extents similar between males and females. Importantly, spontaneous display of these behaviors outside of photostimulation periods as well as serum testosterone levels remained sexually dimorphic, suggesting that ChR2 animals still behaved normally according to their sex without photostimulation. Optogenetic stimulation experiments are “gain of function,” therefore should be interpreted with caution. Nevertheless, one revealing implication extended from these results is that an adult animal still maintains the ability to behave like the opposite sex along with sex-specific patterns and connections in the mPOA and other brain regions. Latent but functional neural circuits that are capable of driving behaviors typically observed in the opposite sex has also been found in female fruit flies^57, 58^, suggesting the possibility of an evolutionarily conserved coding scheme for sexually dimorphic behaviors. Such a neural circuitry scheme can permit stable yet plastic control of sex differences in behaviors amidst motor functions that are largely shared by the two sexes. According to this view, functionality of molecular and anatomical sex differences identified should be viewed more broadly.

While the mPOA targeted in our study is enriched for the expression of *Esr1* and is anatomically separable from other preoptic nucleus such as the ventrolateral preoptic nucleus (VLPO) important for sleep and temperature regulation^59, 60^, one outstanding question remains whether there are subsets of mPOA *Esr1*+ neurons that are specialized and dedicated to the control of male-typical mounting vs. pup retrieval. Noteworthy, the issue of functional heterogeneity prevails across hypothalamic studies^16, 38, 53, 61^. Although results from the catFISH experiment suggest that mPOA *Esr1*+ neurons activated in response to a female or pups are largely distinct, the usage of *c-Fos* transcripts as indicators of neural activities again imposed a limit on the temporal resolution to separate out neurons engaged during appetitive and consummatory social interactions. Future studies using optogenetics assisted single-unit recordings (Opto-tagging)^60^ or GRIN lenses recordings^62^ are required to resolve these issues more precisely. Moreover, as manipulation of mPOA *Esr1*+ as a whole always affected both male-typical mounting and pup retrieval, further partition of this population along the axes of inputs and outputs, genetic markers or neuronal activities using newly developed tools^60, 63–67^ and functional studies of each subset will complete the picture on how mPOA *Esr1*+ neurons regulate the display of behaviors in either sex. Along this line, we will achieve a firmer understanding on how sex-specific wiring in subsets of mPOA *Esr1*+ neurons or changes in their gene expression patterns or excitabilities could render male-typical mating or pup retrieval pathway more or less accessible in one sex vs. the other, thereby lead to sexually dimorphic display of these behaviors. In summary, our study represents an important initial step in efforts to systematically dissect neural control of sexually dimorphic behaviors in vertebrates.

## Methods

### Animals

C57BL/6J animals were purchased from Jackson Laboratory, Slac Laboratory Animal (Shanghai) and bred in house. Esr1-2a-Cre (Esr1^Cre^, #017913) and Vglut2-Ires-Cre (Vglut2^Cre^, #012569) were purchased from the Jackson Laboratory. The Vgat-Cre (Vgat^Cre^) line was previously generated^68^. All Cre lines were bred onto C57BL/6J background for at least one generation. Animals were housed in the Institute of Neuroscience animal facility on 12 h:12 h light/dark cycle with food and water ad libitum. Only heterozygous animals of Cre alleles were used in the study. All experimental protocols were approved by the Animal Care and Use Committee of the Institute of Neuroscience, Chinese Academy of Sciences, Shanghai, China (IACUC No. NA-016-2016).

### Surgery

Adult mice 2–4 months old were anesthetized with isoflurane (0.8–5%) and placed in a stereotactic frame (David Kopf Instrument, Model 1900). Ophthalmic ointment was applied to prevent dehydration. The skull was exposed with a small incision and holes were drilled to inject virus with glass pipettes (15–25 μm in diameter at the tip) and to implant optic fibers. The coordinates for viral delivery into the mPOA were AP, −0.16 mm; ML, ±0.4 mm; DV, −5.150 mm (Paxios and Franklin mouse brain atlas, 2nd edition). 100–400 nl of virus was injected per side at a flow rate of 70 nl per min using a homemade nanoliter injector or at 40 nl per min using a hydraulic pump (Harvard Apparatus). Glass pipettes were left in place for about ~10 min after the injection before retraction. Mono optic fibers (diameter, 200 μm; N.A., 0.37; length, 6 mm; AniLab Software and Instruments Co., Ltd) or dual optic fibers (DFC_200/245–0.37_6.0mm_DF0.9_FLT; Doric Lense) were placed 400 μm above the virus injection site for optogenetic experiments or 50 μm above for fiber photometry recordings. Optic fibers were secured with dental cement and skull screws. Littermates were randomly chosen to be injected with either experimental or control virus. Castration surgeries were performed with animals anesthetized with intraperitoneal (i.p.) injection of ketamine (80 mg kg^−1^) and xylazine (8 mg kg^−1^). Animals were allowed 3–4 weeks to recover after surgeries before subsequent behavioral tests.

### Virus

The following viruses were used in this study: AAVs-hSyn-ChR2-mCherry (Serotype 2/8, titer 2.26 × 10^13^ genomic copies per ml), AAVs-hSyn-mCherry (Serotype 2/8, titer 3.38 × 10^12^ genomic copies per ml), AAVs-Ef1a-DIO-ChR2-mCherry (Serotype 2/8, titer 7.39 × 10^12^ genomic copies per ml), AAVs-Ef1a-DIO-mCherry (Serotype 2/8, titer 8.93 × 10^12^ genomic copies per ml), all purchased from Obio Technology Co. (Shanghai); AAVs-Ef1a-DIO-EYFP (Serotype 2/9, titer 8.6 × 10^12^ genomic copies per ml), AAVs-hSyn-GCaMP6s (Serotype 2/9, titer 8.8 × 10^12^ genomic copies per ml), AAVs-hSyn-EGFP (Serotype 2/9, titer 4.52 × 10^12^ genomic copies per ml); AAVs-hSyn-DIO-GCaMP6s (Serotype 2/9, 3.85 × 10^12^ genomic copies per ml), AAVs-hSyn-DO-GCaMP6s (Serotype 2/9, titer 1.18 × 10^13^ genomic copies per ml), AAVs-CAG-DIO-GtACR1-P2A-EGFP (Serotype 2/5, titer 7.5 × 10^12^ genomic copies per ml), all produced by Shanghai Taitool Bioscience, Co. Ltd; and AAVs-Ef1a-DIO-taCasp3.Tevp (Serotype 2/5, titer 4.2 × 10^12^ genomic copies per ml) purchased from University of North Carolina Gene Therapy Center VectorCore.

### Behavioral assays

All behavioral tests were initiated at least half an hour after the onset of the dark cycle, and were recorded using an infrared camera at a frame rate of 25 Hz and scored by experimenters blinded to the genotype, group information, or photostimulation status of the animals involved. All animals tested for behaviors, except for those used in optogenetic inhibition experiments, were naive virgin mice prior to the test. In optogenetic inhibition experiments, all male mice were co-housed with female mice and some of them became fathers before testing while some females were co-housed with pups, or mated to become mothers, or treated with weeks of testosterone injection before testing. Except for animals used in optogenetic activation experiments, all animals were singly housed 3–7 days before behavioral testing.

For mating behavior tests, a hormonal primed ovariectomized C57BL/6 female was introduced to the home cage and videotaping for 30 min. Mating behavior tests were repeated three times with different stimulus animals at least three days apart. Parental behavior was tested by scattering three pups age between P1 and P4 at the edge away from the nest and videotaping the animal for ~15 min and were repeated 2–3 times. Territorial aggression was tested by introducing an intruder mouse of Balb/c stain purchased from Slac Laboratory Animal (Shanghai) to the home cage of the tested animal and videotaping for ~15 min.

Videos were manually annotated on a frame-by-frame basis using a custom-written MATLAB program as previously described^8^. Behaviors were scored according to the following criteria: nose-to-face, nose-to-body, or nose-to-urogenital contacts initiated by the tested animals are scored collectively as “social investigation,” of which nose-to-urogenital contact is specifically defined as “chemoinvestigation,” also known as “sniff.” “Mount” is scored when the experimental animal places its forelimbs on the back of the stimulus, climbs on top and moves the pelvis. Rhythmic pelvic movements after mount are scored as “Pelvic thrust.” Actions initiated by the resident toward a male intruder including lunges, bites, tumbling are scored as “Attack.” “Pup contact” is scored when an animal contacted a pup with its nose or mouth. “Pup retrieval” is scored when an animal holds the pup with its mouth and traveled, usually toward the nest. “Crouching” is scored when the animal arches its back and hovers over pups in the nest. Pups were always inspected after behavioral trial for possible wounds. About 2% of behavioral trials resulted in wounded pups. Exclusion of these trials did not influence any conclusion.

### Optogenetic activation

Animals used for optogenetic experiments were group housed. On the day of the behavioral test, they were introduced to a fresh cage. An external optic fiber was used to connect a 473 nm laser power source (Shanghai Laser and Optics Century Co. or Changchun New Industries Optoelectronics Tech Co., Ltd.) to the implanted optic fiber in the animal. The external optical fiber was attached to a rotary joint (FRJ_1 × 1_FC_FC, Doric Lense) to allow the animal to freely behave. The tested animal was allowed to explore the cage for ~15 min with the external fiber attached. Afterward, a custom MATLAB program was started to control a Master 9 (A.M.P.I.), which sent a trigger to initiate recording from the camera and signals to the laser to deliver 15 s of photostimulation at 40 Hz 12 mW or 20 Hz 5 mW, spaced 90–120 s apart at random. The laser power was adjusted for each animal according to the luminance transitivity efficiency of the implanted optical fiber measured prior to implantation to assure the power of the light emitted at the tip of the fiber. During laser stimulation, animals were either tested alone for locomotion, or with a hormonal primed ovariectomized female, a C57BL/6 male, a young rat age of P13–P15, pups age of P1–P4, or rubber blocks of similar size of pups as the stimulus. Five–twelve stimulations were given during each assay. One–four assays with each stimulus (3–5 min apart) were performed on any given day for each animal. After completion of all behavioral testes, animals were given trains of photostimulation (15 s, 12 mW, 40 Hz, 10 times, a fixed 105 s interval) and perfused transcardially with 4% paraformaldehyde one hour after light stimulation for histological analysis. Blood was also collected at the time of killing for hormonal measurements.

### Fiber photometry

Animals used for fiber photometry experiments were singly housed 3–7 days before behavioral testing. During the day of testing, the implanted optic fiber was connected to F-scope (Biolink Optics Technology Inc., Beijing), an integrated fiber photometry recording setup, through an external optical fiber. In the F-scope, a 488 nm excitation laser (OBIS 488LS; Coherent) is reflected off a dichroic mirror (MD498, Thorlabs). The emission signals collected through the implanted optic fiber are filtered with a bandpass filter (MF525-39, Thorlabs) and directed onto a photomultiplier tube (PMT, R3896, Hamamatsu). During recordings, the laser power was adjusted for each animal according to the luminance transitivity efficiency of the implanted optical fiber to assure that light emitted at the tip of fiber was ~30 μw and the PMT voltage gain was set to 500 v for GCamp6s animals and 300–350 v for control animals. Once started, the F-scope software sent a trigger signal to initiate video recording from the infrared camera. Emission signals were low-pass filtered at 30 Hz and were sampled at 500 Hz with a data acquisition card (USB6009, National Instrument) using a software provided by the Biolink Optics. For a given trial, animals were first allowed to explore for ~5 min, during which time baseline signals were recorded. Afterward, a stimulus such as a hormonal primed ovariectomized female, a fake plastic mouse toy, pups age of P1–P4 or rubber blocks of similar size of pups were introduced. Animals were allowed to interact and behavior with the stimuli for 15–90 min before removal of the stimuli, after which signals were recorded for another ~5 min.

### Optogenetic inhibition

Animals used for optogenetic inhibition experiments were single housed. Male mice were co-housed with females to be sexually experienced or to become fathers before testing. Virgin female mice were tested as naive animals for male-typical mating and for maternal behaviors. In cases of poor baseline maternal behaviors during the initial test, they were co-housed with pups overnight and tested again for maternal behaviors. After testing of maternal behaviors, some females were treated every other day with subcutaneous injection of testosterone (100 μg in 50 μl sunflower oil, Shanghai Pharm.) for about 3 weeks before being tested again for male-typical mating. Females tested as mothers were mated after viral injection to produce their own pups.

Before the behavioral test, a dual fiber-optical patch cord (DFP_200/230/900–0.37_1m_DF0.9_2FCM, Doric Lense) was used to connect a 473 nm laser power source (Shanghai Laser and Optics Century Co. or Changchun New Industries Optoelectronics Tech Co., Ltd.) to the implanted dual optic fiber (DFC_200/245–0.37_6.0mm_DF0.9_FLT, Doric Lense) in the animal via a rotary joint (FRJ 1 × 2i FC-2FC 0.22, Doric Lense) to allow the animal to behave freely. After ~10 min of habitation, the camera was triggered to record the behavioral process by a signal sent from Master 9, which was controlled by a custom written MATLAB program. To deliver light after approaching, light (~12 mW) was automatically triggered to be continuously delivered as long as the positions of the tested animal were detected by the program to be within one body length to the hormonal primed ovariectomized female in mating behavior tests or within the pup region, which was defined by marking a slight larger area surrounding each scattered pup in maternal behavior tests. At least two light trials and two no light trials were performed alternatively for each test. To deliver lights after initiation of defined behaviors, blue light (~12 mW) of fixed length (5 or 10 s) was manually triggered when the experimenter observed the behavior of interests in real-time. One or two light trials were performed alternatively for each test.

### Fluorescent immunostaining

Animals were anesthetized with 10% chloral hydrate and transcardially perfused with PBS followed by ice-cold 4% paraformaldehyde (PFA) in PBS. Afterward, brains were sectioned at 40 μm thickness using a vibratome (VT1000S, Leica). Sections were divided equally into several sets and processed for staining or mounted directly. Brain sections were blocked in 5% goat serum in AT (0.1% Triton and 2 mM MgCl_2_ in PBS) for 1 h at room temperature and incubated overnight at 4 °C in AGT (0.5% normal goat serum, 0.1% Triton and 2 mM MgCl_2_ in PBS) containing appropriate primary antibody (rabbit anti-c-Fos, 1:2000, Santa Cruz, Cat# sc‐52; rabbit anti-Esr1^54^, 1:10,000, Millipore, Cat# 06-935). The next day, brain sections were washed with AGT for three times (30 min each) and incubated with appropriate secondary antibodies (Alexa Fluor 488 conjugated, 1:1000; Cy3 conjugated, 1:1000, Jackson ImmunoResearch) at room temperature for 2 h. Brain sections were counterstained with Neuro Trace (Life Technologies, Cat# N21479, 1:300) or DAPI (5 mg ml^−1^, 1:1000, Sigma) in AT. After several washes in AGT, AT and PBS, brains sections were mounted onto glass slides.

### DAB staining

Brain sections were prepared similar to fluorescent immunostaining experiments and were pretreated with 3% H_2_O_2_ for 30 min at room temperature to block the endogenous peroxidase, washed twice for 10 min each in PBST (0.3% Triton X-100 in PBS), blocked with 5% goat serum in PBST for 2 h at room temperature, and incubated with primary anti-c-Fos antibody (Rabbit anti-c-fos, 1:2000, Santa Cruz, Cat# sc‐52) in PBST overnight at 4 °C. The next day, sections were washed six times in PBST, 10 min each, and then incubated with the biotin-conjugated goat anti-rabbit secondary antibody (Cat#111-065-003, Jackson ImmunoResearch) in PBST for 2 h at room temperature. After three washes in PBS, 5 min each, brain sections were stained with VECTASTAIN^®^ ABC Reagent for 30 min following the manufacturer’s manual. After two 5-min washes in PBS, brain sections were incubated in 3,3-diaminobenzidine (Cat# D5637-5G, Sigma) solution with nickel intensification until achieving desired staining intensity. Reaction was stopped by rinsing sections in tap water. Brain sections were mounted onto glass slides and captured under ×4 or ×10 objectives with conventional light microscopes.

### In situ hybridization

DNA templates for generating in situ probes were cloned using the following primer sets for each corresponding gene: *Vgat*, 5′-gccattcagggcatgttc-3′ and 5′-agcagcgtgaagaccacc–3′; *Vglut2*, 5′-atcgactagtccaaatcttacggtgctacctc-3′ and 5′-atcgctcgagtagccatctttcctgttccact-3′; *Cre*, 5′- ccaatttactgaccgtacacca-3′ and 5′-tatttacattggtccagccacc-3′; *GAD1*, 5′-cattgaggagatagagaggttg-3′ and 5′-agagaagagcgaaggctact-3′; *Galanin*, 5′-atcctgcactgaccagcc-3′ and 5′-ttggcttgaggagttggc-3′. Anti-sense RNA probes were transcribed with T7 RNA polymerase (Promega, Cat# P207E) and digoxigenin (DIG)-labeled nucleotides. Animals were anesthetized with 10% chloral hydrate and transcardially perfused with DEPC-treated PBS (D-PBS) followed by ice-cold 4% paraformaldehyde (PFA) in D-PBS. Afterward, brains were sectioned at 40 μm thickness using a vibratome (VT1000S, Leica). Brain sections were washed in 2XSSC buffer containing 0.1% triton for 30 min, acetylated in 0.1 M triethanolamine (pH 8.0) with 0.25% acetic anhydride (vol/vol) for 10 min, equilibrated in pre-hybridization solution for 2 h at 65 ℃ and subsequently incubated with 0.5 μg ml^−1^ of specific RNA probes in hybridization buffer overnight at 65 ℃. The next day, sections were rinsed in pre-hybridization solution and pre-hyb/TBST (TBS with 0.1% tween-20) for 30 min each. Next, sections were washed with TBST for twice and TAE for three times, each for 5 min. Sections were then transferred into wells in 2% agarose gel, which were run in 1XTAE at 60 V for 2 h to remove unhybridized probes. Sections were then washed twice in TBST, and subsequently incubated with sheep anti-digoxygenin-AP (1:2000, Roche, Cat# 11093274910), sometimes together with rabbit anti-Esr1 antibody (1:3000, Millipore, Cat# 06–935) for co-staining purposes, in 0.5% blocking reagent (Roche, 11096176001) at 4 °C overnight. On the second day, for brightfield staining sections were washed and stained with NBT (Roche, Cat# 11383213001) and BCIP (Roche, Cat# 11383221001) for 4–10 h at 37 ℃. For fluorescent in situ hybridization combined with immunohistochemistry, sections were stained with fluorescent secondary antibodies first, followed by staining with fast red (HNPP Fluorescent Detection Set, Roche, Cat# 11758888001) and counterstaining with DAPI (5 mg ml^−1^, 1:1000, Sigma) in PBS. All sections were washed after staining and mounted on glass slides. Images were captured with ×20 objective using a conventional or confocal microscope.

### catFISH

Most animals used in the catFish experiment were experienced. For the procedure, animals were allowed two 5-min episodes of social interactions with a hormonal primed ovariectomized female or scattered pups, 30 min apart. Immediately after the second episode, animals were anesthetized with 10% chloral hydrate and transcardially perfused with DEPC-PBS followed by ice-cold 4% PFA in PBS. Brains were dissected and post-fixed over night at 4 °C and dehydrated with 30% sucrose in DPEC-PBS. Afterward, brains were sectioned at 20 μm thickness and mounted to SuperFrost Plus^®^ Slides (Fisher Scientific, Cat. No. 12-550-15). After drying in the air, slides were stored in −80 °C before being processed according to RNAscope^®^ Multiplex Fluorescent Reagent Kit v2 User Manual (ACD Bio.). Probes against *c-Fos* intron, *c-Fos* mRNA, and *Esr1* were ordered from ACD Bio. and used in the experiment. Images were captured under a ×60 objective using a confocal microscope.

### Electrophysiological recordings

C57BL/6 animals injected with AAVs encoding ChR2-mCherry in the mPOA or Esr1^Cre^ mice injected with AAVs encoding GtACR1 were anesthetized with isoflurane and transcardially perfused with ice-cold oxygenated (95% O_2_/5% CO_2_) high-sucrose solution (composition in mM: 2.5 KCl, 1.25 NaH_2_PO4, 2 Na_2_HPO4, 2 MgSO_4_, 213 sucrose, 26 NaHCO_3_). After dissection of the brain, coronal sections including the mPOA were cut at 250 μm using a vibratome (VT-1200S, Leica) in ice-cold oxygenated cutting solution. Brain slices were then incubated in artificial cerebrospinal fluid (ACSF; composition in mM: 126 NaCl, 2.5 KCl, 1.25 NaH_2_PO_4_, 1.25 Na_2_HPO_4_, 2 MgSO_4_, 10 glucose, 26 NaHCO_3_, 2 CaCl_2_) at 34 °C for at least 1 h and recorded at room temperature. Cells were identified under fluorescent microscope and visualized by infrared differential interference contrast (BX51, Olympus). Whole-cell current clamp recordings were accomplished with a MultiClamp700B amplifier and Digidata 1440A interface (Molecular Devices). The patch-clamp electrode (5–8 MΩ) was backfilled with an intracellular solution (composition in mM: 120 K‐gluconate, 4 KCl, 10 HEPES, 10 sodium phosphocritine, 4 Mg‐ATP, and 0.3 Na_3_‐GTP, pH:7.3, 265 mOsm). For optogenetic activation, blue light (473 nm, 10 ms width, 14 mw mm^−2^, 40 pulses) was delivered onto slices through ×40 objective using an X-Cite LED light source (Lumen Dynamics). The spike fidelity in ChR2-expressing neurons was measured by counting the number of light pulses that successfully evoked action potentials at different frequencies. Spike fidelity was averaged across five stimulations and plotted. For optogenetic inhibition, the action potential of GtACR1-expressing neurons was induced by withholding the membrane potential −48 mW with a current injection and repeated continuous blue light was delivered onto slices.

### Calcium imaging in brain slices

AAVs encoding ChR2 and AAVs encoding GCamp6s, both driven by hSyn, were co-injected into the mPOA of C57BL/6 animals. Acute brain slices including the mPOA were prepared similar to the electrophysiological procedures described above. Calcium imaging was carried out using two photon laser-scanning microscope (Ultima, Prairie Instruments Inc.) with a 20X/0.95-NA XLUMPLFL water-immersion objective (Olympus). The Ti:sapphire laser was tuned to 940 nm, and images of size of 512 × 512 pixel were acquired through 525/70 nm emission filter at 1.2 Hz frame rate. Randomized trains of 470 nm light (10-ms pulse, 15 s, 8.4 mW mm^−2^) of different frequencies (10 Hz, 20 Hz, 40 Hz) were delivered to the slices with intervals of ~2 min. Images were analyzed using ImageJ.

### Hormone assays

Trunk blood was collected at the time of killing before perfusion. Serum was prepared. Hormone titers were assayed using a testosterone ELISA kit (DRG Instruments GmbH, Germany, Division of ARG international, Inc, Cat# EIA-1559) according to the manufacturer’s protocols.

### Data analysis

All codes were written in MATLAB and used for data analysis, and are available upon request. No sample size calculation was performed. The number of cells recorded in slice or counted in staining and the number of animals used for behavioral tests and staining were chosen according to the published literature of similar experiments. Data points from animals which upon post hoc histological analysis were deemed as mis-hits according to pre-established criteria were excluded from the analysis. In all, three female Esr1^Cre^ mice and one male Esr1^Cr*e*^ mouse injected with GtACR1, one male and female Vgat^Cre^ and one female Esr1^Cre^ mouse injected with Casp3 were excluded from analysis.

To analyze data from Ca^2+^ imaging experiments in brain slices, images were quantified in ImageJ. Briefly, GCaMP6s+ cells were outlined manually and the relative fluorescence changes (Δ*F*/*F*) were calculated for each cell with the equation Δ*F*/*F* = (*F*−*F*_0_)/*F*_0_, in which *F*_0_ is the average pixel intensity in the last 10 frames before the photostimulation and *F* is the average pixel intensity in the first 6 frames after the photostimulation. Images acquired during the photostimulation periods were excluded from the analysis. An activated cell was operationally defined as cells showing increase in Δ*F*/*F* that was 5 standard deviations away from average Δ*F*/*F* values measured in 30 frames before the first photostimulation.

To analyze light-evoked locomotion, motion traces were first extracted using custom MATLAB codes, which recognize the centroid of an animal. Motion speed was then calculated in 1 s time bins by measuring the distance traveled. The 15 motion speed data points during a photostimulation period were compared with previous 15 points to test whether that light stimulation induced speed increase in that trial. At the animal level, averages of motion speeds during each photostimulation period were compared with averages of motion speeds immediately prior to each photostimulation period to determine whether light-induced locomotion increases in that animal. For optogenetic-induced mounting and pup retrieval, videos were first scored blindly. Video logs were then aligned to photostimulation logs to extract the trial percentage, the onset latency and the total duration of evoked behaviors. Only behaviors that occurred during light stimulation were counted as optogenetically induced behaviors. If a behavior occurred prior to but nevertheless ran into a photostimulation period, such behavior was not counted as optogenetically induced behaviors. Parameters were always averaged across photostimulation periods in the same behavioral trial and then across trials if there were multiple. To plot time distribution of optogenetically evoked behaviors, only photostimulation periods in which behaviors occurred were used to average. In behavioral trials with a male, a young rat or fake pups were used as the stimulus or when ChR2 animals were castrated, optogenetically induced behaviors were compared with spontaneous behaviors that occurred in the 15 s period immediately preceding photostimulation periods.

To analyze the effect of optogenetically inhibition on specific behavior, videos were first scored blindly. Video logs were then aligned to photostimulation logs to extract behavior events that encountered light stimulation. To plot various parameters of specific behaviors, behavioral events encountered light stimulus were averaged on the trial level if multiple trials existed and then on the animal level. To plot the accumulation distribution of specific behaviors, only behavior events that encountered light stimulation were analyzed.

To simulate an activation center for a ChR2 animal, a 1100×1500 μm area with one side close to the midline and the perpendicular side close to the bottom of the brain was cropped from each coronal brain section and was resized to 1 μm per pixel. c-Fos signals in each 100×100 μm were extracted semi-automatic using MATLAB program along with ImageJ and summed into a 11 × 15 matrix according to their locations within each section. The average number of NeuN/HuCD+ cells within a 100 μm^2^ square was estimated to be ~25. To calculate the coordinates of the activation center along the lateral–medial and dorsal–ventral axis, numbers within the 11 × 15 matrix were averaged across a series of brain sections that spanned anteriorly and posteriorly for each animal and fitted with two-dimension Gaussian function in MATLAB. To calculate the anterior–posterior coordinate of the activation center, the total c-Fos number was summed for each brain section and fitted with a single Gaussian function.

For fiber photometry experiments, raw fluorescence signals were further adjusted according to the overall trend to account for photo bleaching. Trials with jitter, square waves of signals or of extremely low signal to noise ratio were excluded from further analysis. For initial response, the values of fluorescence change (Δ*F*/*F*) was derived by calculating (*F*−*F*_0_)/*F*_0_, where *F*_0_ is the median of the baseline fluorescence signal. For the fluorescence signals aligned to behaviors, data were segmented based on behavioral events within individual trials and average signals 10–15 s before behavior initiation were used as *F*_0_. To analyze the statistical significance of the two event-related fluorescence signals, *t* test with false discovery rate (FDR) of 0.05 was used. To correlate Ca^2+^ transients and behaviors, a ramping Ca^2+^ event was counted when the Δ*F*/*F* values were 3 standard deviation above the baseline and lasted for longer than 2 s. The numbers of Ca^2+^ events were counted between the first and last behavior and correlated with the numbers of behavioral events during that period. Only behavioral trials that had more than two mounts and more than one retrieval were used for correlation analysis.

Histological images were captured with ×20 objective on a confocal microscope unless otherwise specified. Images were processed and counted in ImageJ with custom-written MATLAB codes. All counting was done by experimenters blind to the sex or the test condition of the animal. To analyze the viral expression of ChR2, the co-labeling of ChR2, c-Fos and Nissl in center injection area was counted manually. To analyze co-labeling of Esr1 and *Vglut2*, *Vgat* or *Galanin*, five relatively fixed areas in five brain sections of similar positions were selected to count manually. To test the specificity of Cre expression in Esr1-cre animals, the co-labeling of Esr1 and *Cre* was counted manually in various areas of multiple slices obtained from one animal. To quantify the images of catFISH experiments, which were taken under a ×60 objective, cells positive for cytoplasmic or nuclear *c-Fos* and for *Esr1* were recognized with DAPI as the counterstaining and counted manually. To quantify the effects of viral ablation of Esr1+ neurons, Esr1+ signals in the mPOA as defined by the Allen Brain Atlas were counted for both sides using unbiased stereology with Stereo Investigator software (MBF bBioscience). Specifically, using an optical fractionator probe, Esr1+ signals were enumerated in a 30×30 μm counting box within a 100×100 μm sampling grid. To quantify the effects of viral ablation of *Vgat*+ or *Vglut2*+ neurons, brain sections were imaged with ×10 objective via Olympus VS120. Areas that stained with *Vgat*+ or *Vglut2*+ signals within the mPOA and adjacent regions were extracted semi-automatic using MATLAB codes.

### Statistics

For bar graphs, data are presented as mean ± s.e.m. In box and whiskers plots, data are presented as min to max whiskers and the mean indicated by “+” and the median indicated by a horizontal line. Unless specified otherwise, the following statistical tests were performed and all tests were specified as two-sided. Categorical data were analyzed by Fisher’s exact test. Paired data were analyzed by paired test. To analyze the statistic difference of accumulation distribution, two-sample Kolmogorov–Smirnov test was used. Data with one independent variables were analyzed by one-way ANOVAs followed by post hoc test with Bonferroni correction. Data with two independent variables were analyzed by two-way ANOVAs followed by post hoc test with Bonferroni correction. For other comparisons, data were tested for the distribution with Lilliefors’ goodness-of-fit normality test. If data passed the normality test, parametric test (Student’s *t* test) was used. Otherwise, nonparametric Wilcoxon rank sum test was used. The *p* value and the correlation coefficient for Pearson’s correlation were computed by using a Student’s *t* distribution for a transformation of correlation. *p < 0.05, **p ≤ 0.01, ***p < 0.001.

### Data availability

All data in this study are available on request.

## Electronic supplementary material

Supplementary Information

Description of Additional Supplementary Files

Supplementary Movie 1

Supplementary Movie 2

Supplementary Movie 3

Supplementary Movie 4

Supplementary Movie 5

Supplementary Movie 6

Supplementary Movie 7

Supplementary Movie 8

Supplementary Movie 9

Supplementary Movie 10

Supplementary Movie 11
