# Supplementary material for: Medial preoptic area in mice is capable of mediating sexually dimorphic behaviors regardless of gender
Source: Nat Commun. 2018 Jan 18;9:279. doi: 10.1038/s41467-017-02648-0 (PMC5773506; doi:10.1038/s41467-017-02648-0)
Supplement: Supplementary file 1 — Supplementary Information [file 41467_2017_2648_MOESM1_ESM.pdf]

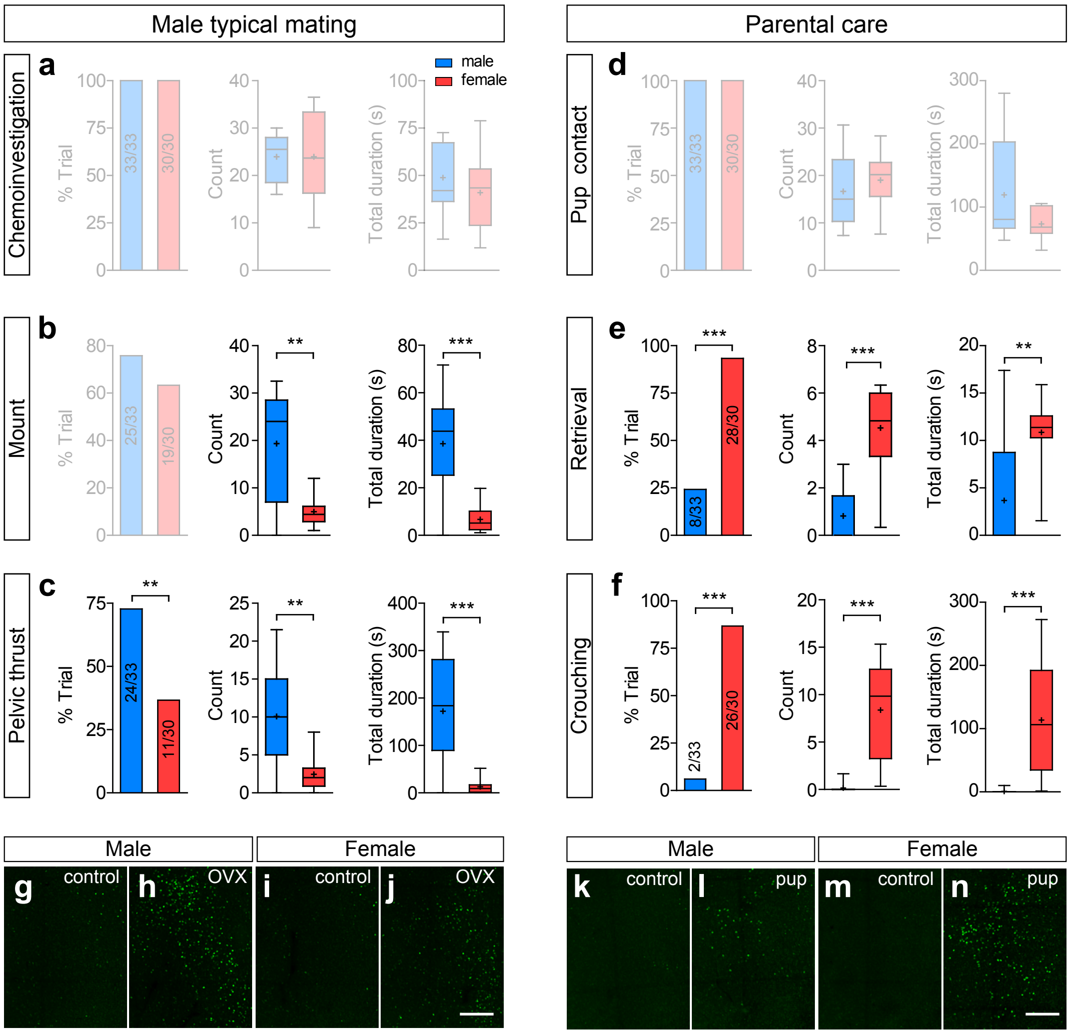

Supplementary Figure 1. Sexual dimorphic display of male-typical mating and parental care in virgin C57BL/6 mice. a-c. Sexual dimorphic display of male-typical mating behavior in virgin C57BL/6 mice. When given a hormonal primed ovariectomized female (OVX) as the stimulus, the percentage of trial (left), count (middle) and total duration (right) of chemoinvestigation were not different between the two sexes (a), but count and total duration of mount (b) and pelvic thrust (c) were higher in males. **d-f.** Sexual dimorphic display of parental care in virgin C57BL/6 mice. When given scattered pups as the stimuli, the percentage of trial (left), count (middle), and total duration (right) of pup contact was not different between the two sexes (d), but all parameters of pup retrieval (e) and crouching (f) were higher in females than males. N=11 male and 10 female. **g-j.** Representative c-Fos immunohistochemistry staining in the mPOA of control (no stimulus) and experimental animals, which were sacrificed one hour after exposure to an OVX as the stimulus. **k-n.** Representative c-Fos immunohistochemistry staining in the mPOA of controls (no stimulus) and experimental animals, which were sacrificed one hour after exposure to pups as the stimuli. Scale bar, 200μm. Fisher's exact test for categorical data, unpaired t-test or Wilcoxon rank sum test otherwise. ** $p < 0.01$, *** $p < 0.001$.

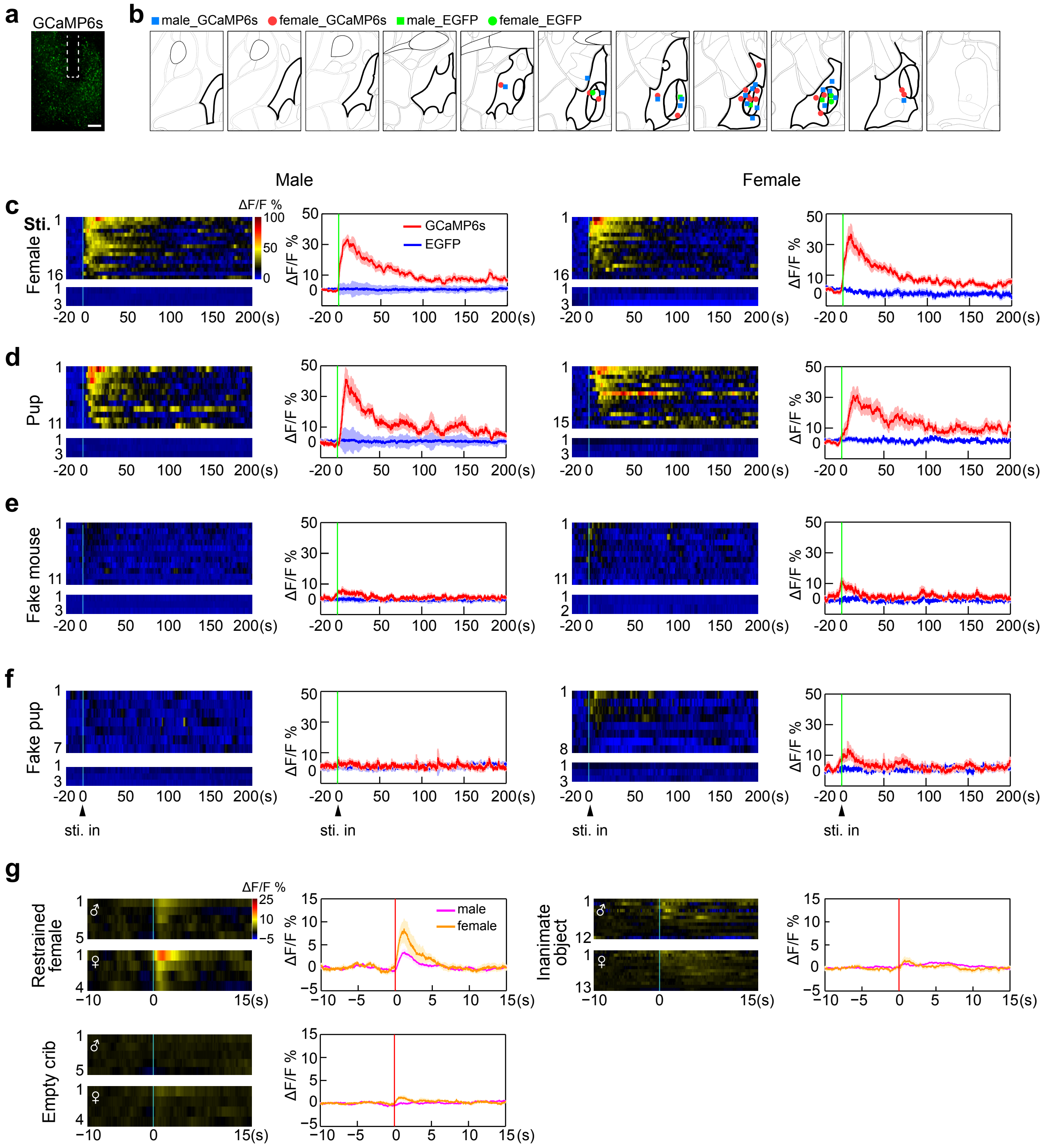

Supplementary Figure 2. Response of the mPOA to different stimuli. a. An example image from a wild type animal showing expression of GCaMP6s and the placement of the optic fiber (dashed lines). Scale bar, 200μm. **b.** Recording sites of GCaMP6s and control animals of both sexes were projected on the Allen reference atlas (http://www.brain-map.org/, image 49-59) with bold lines highlighting the region that corresponds to the mPOA. **c-f.** ΔF/F signals in heatmap (left) and quantification plots (right) for male and female of experimental (top heatmap) and control groups (bottom heatmap) when female (c), pup (d), fake mouse (e), or fake pup (f) were given as the stimuli. Time "0" is set to the introduction of the stimulus. Scale bar in panel c applies to all heatmaps in c-f. Lines indicate mean values and shaded area s.e.m.. **g.** Heatmap representations of ΔF/F signals around social investigations towards a female restrained under a crib, or investigations towards an inanimate object, or investigations towards an empty crib in males and in females with time "0" aligned to the onset of the behavior. Scale bar applies to all heatmaps in this panel. Quantification of ΔF/F signals associated with investigation is shown on the right with lines indicating mean values and the shaded area s.e.m..

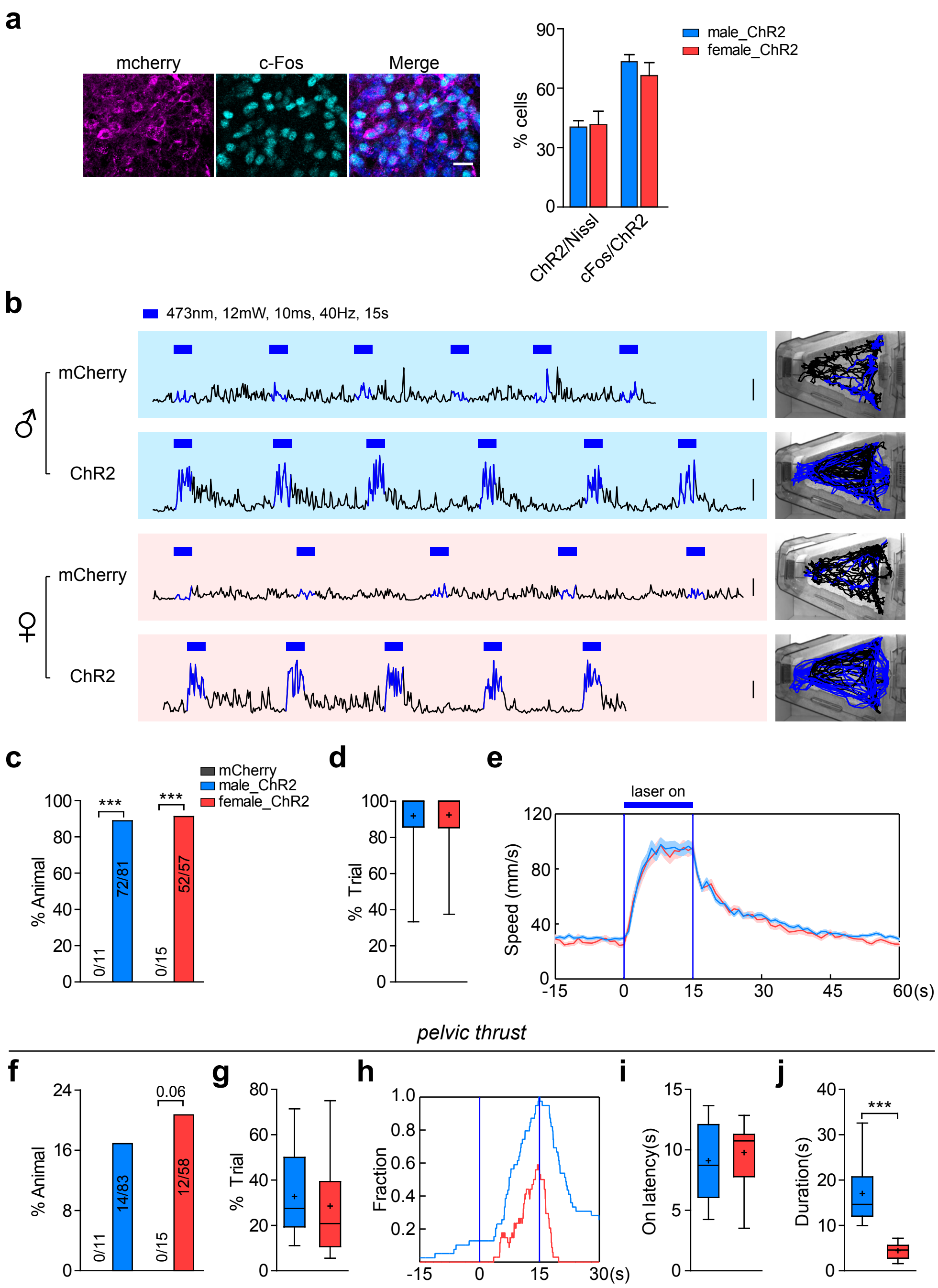

Supplementary Figure 3. Optogenetically induced locomotion increase and pelvic thrust in ChR2 animals. a. Representative images showing expression of ChR2 as indicated by mCherry signal and c-Fos after light stimulation on the left and quantification on the right. N=6 male and 5 female. Scale bar, 20μm. **b.** On the left, representative plots of locomotion speed of mCherry and ChR2 animals of either sex with blue bars indicating light stimulation. On the right, representative plots of movement traces with blue and black lines denoting data points during light stimulation and baseline respectively. Scale bar, 100mm/s. **c-e.** Quantification of optogenetically induced locomotion increase. c. ChR2 but not control animals displayed locomotion increase during photostimulation. d. Box plots of trial-by-trial occurrence of light induced locomotion increase for each ChR2 animal. e. Average distribution of light induced locomotion before, during and after laser stimulation. N= 81 ChR2 and 11 mCherry for male and 57 ChR2 and 15 mCherry for female. **f-j.** Optogenetic activation of the mPOA elicited male-typical pelvic thrust towards hormonal primed ovariectomized females. f. The percentage of animals that displayed pelvic thrust behavior during photostimulation was not different between the two sexes. g. Box plots of trial-by-trial occurrence of light induced pelvic thrust behavior for ChR2 animals. h. Average distribution of light induced pelvic thrust behavior during laser stimulation. i. Box plots of onset latency of light induced pelvic thrust behavior for ChR2 animals. j. Box plots of duration of pelvic thrust, which was significantly longer in ChR2 males than females. N= 83 ChR2 and 11 mCherry for male and 58 ChR2 and 15 mCherry for female. Fisher's exact test for categorical data, unpaired t-test or Wilcoxon rank sum test otherwise. *** $p < 0.001$.

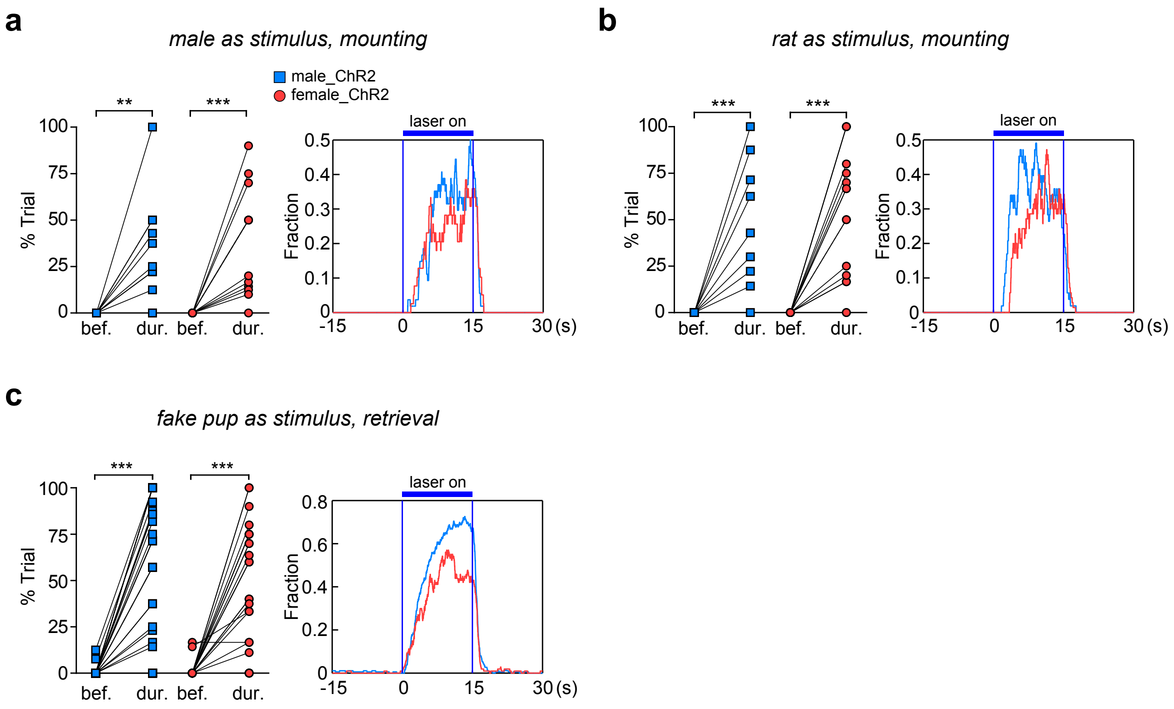

Supplementary Figure 4. Optogenetically induced behaviors towards male and young rat and fake pups. a-b. Occurrence and distribution of mounting behavior before (bef.), during (dur.) and after light stimulation when a male mouse (a) or a young rat (b) was used as the stimulus. N=13 male and 13 female for panel a, and 9 male and 16 female for panel b. **c.** Occurrence and distribution of retrieval behavior when fake pups were given as the stimuli. N=46 male and 27 female. Paired t-test. ** $p < 0.01$, *** $p < 0.001$.

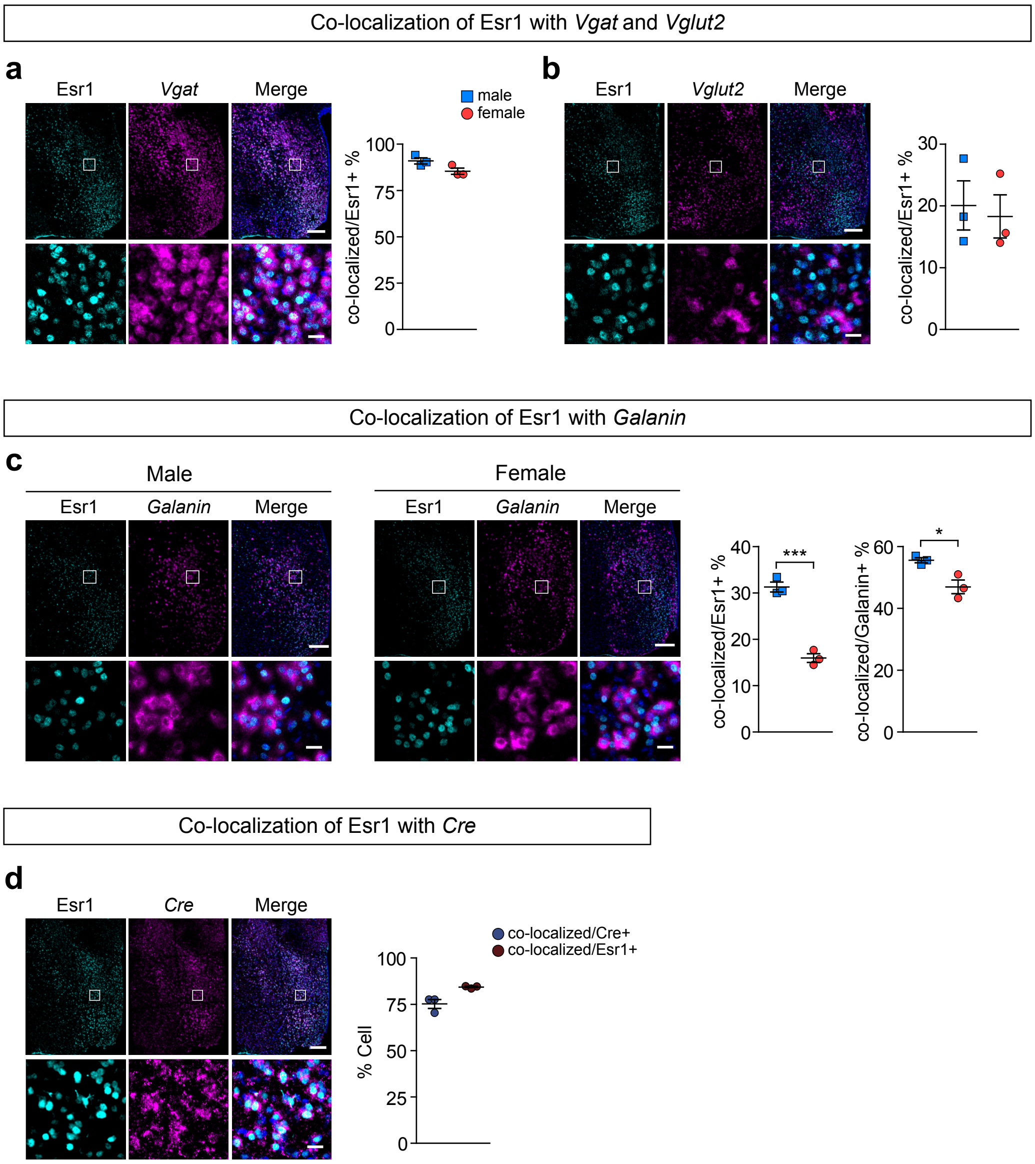

Supplementary Figure 5. Characterization of mPOA Esr1+ neurons and the expression of *Cre* in Esr1^{Cre} animals. a-c. Dual fluorescent staining of Esr1 protein and *Vgat* (a) or *Vglut2* (b) or *Galanin* (c) mRNA showed overlapping of expression. Quantification shown on the right. N=3/sex/condition. **d.** Dual fluorescent staining of Esr1 protein and *Cre* mRNA in a Esr1Cre female showed overlapping of expression. Quantification of three windows of view shown on the right. Scale bar, top images 200μm, bottom images 20μm. Unpaired t-test. * p< 0.05, *** p < 0.001.

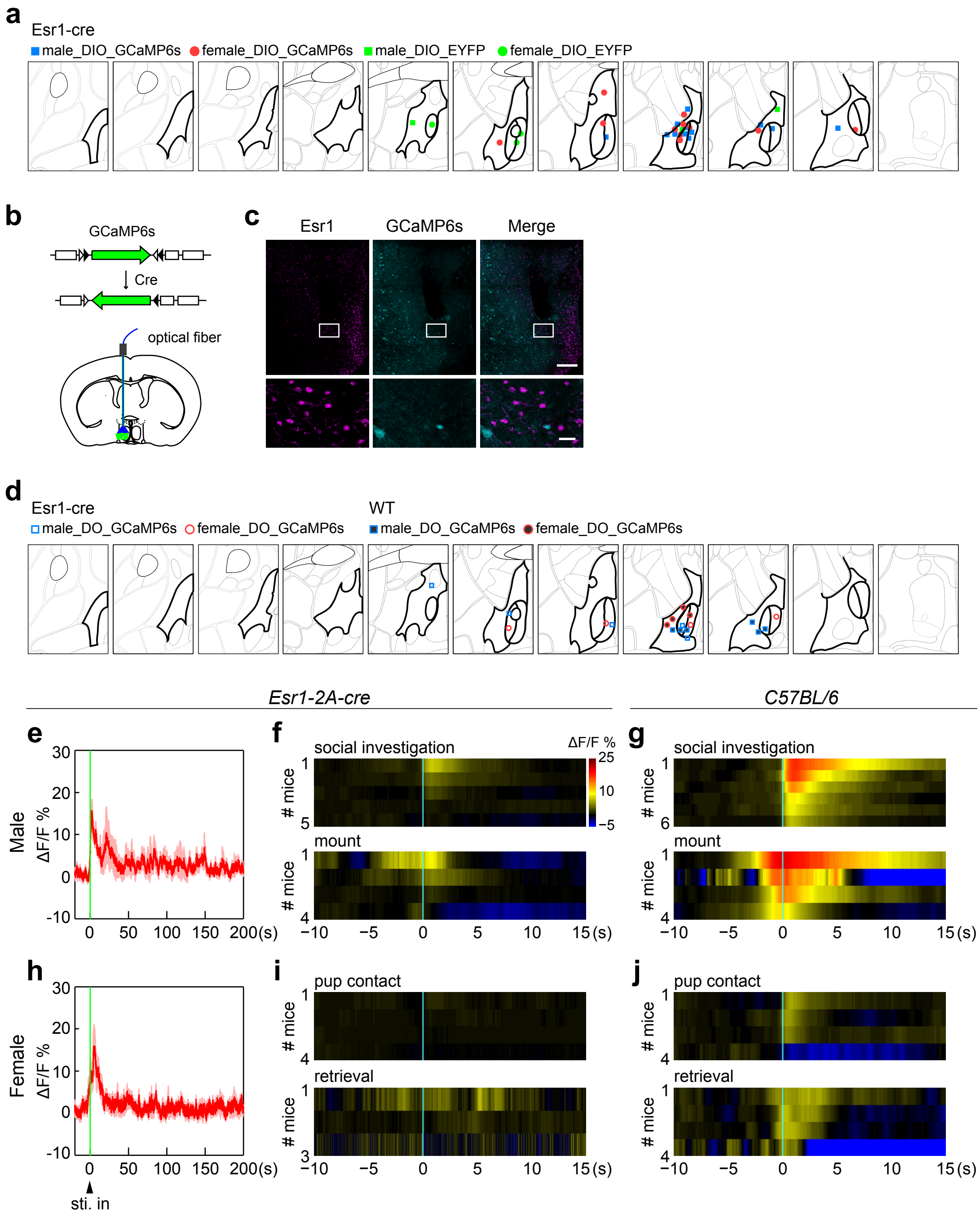

Supplementary Figure 6. Activities of mPOA *Esr1+* and *non-Esr1+* during social interactions. a. Recording sites of Esr1^{Cre} animals of both sexes injected with Cre-dependent GCaMP6s or EGFP virus projected on the Allen reference atlas (http://www.brain-map.org/, image 49-59) with bold lines highlighting the region that corresponds to the mPOA. N=12 male and 11 female for GcaMP6s group and 3 male and 3 female for the EGFP group. **b.** Strategy to record activities from mPOA *non-Esr1+* neurons. AAVs encoding DO-GCamp6s driven by hSyn were injected into Esr1^{Cre} and wild type C57BL/6 animals. **c.** Representative images showing GCamp6s expression (cyan) not co-localized with Esr1 immunostaining signals (magenta) in Esr1^{Cre} animals injected with the virus. Images on the bottom depict in higher magnitude white boxes in images on the top. Scale bar, 200μm for top images and 30μm for bottom images. **d.** Recording sites of Esr1^{Cre} and wildtype animals of both sexes injected with DO-GCaMP6s. N= 5 male and 4 female for Esr1^{Cre} group and 6 male and 4 female for wildtype animals. **e-g.** ΔF/F signals in Esr1^{Cre} and wildtype males injected with the virus upon initial encounter with a female stimulus and around social investigation and mount. Scale bar in panel f applies to all heatmaps in this figure. **h-j.** ΔF/F signals in Esr1^{Cre} and wildtype females injected with the virus upon initial encounter with pups and around pup contacts and retrieval.

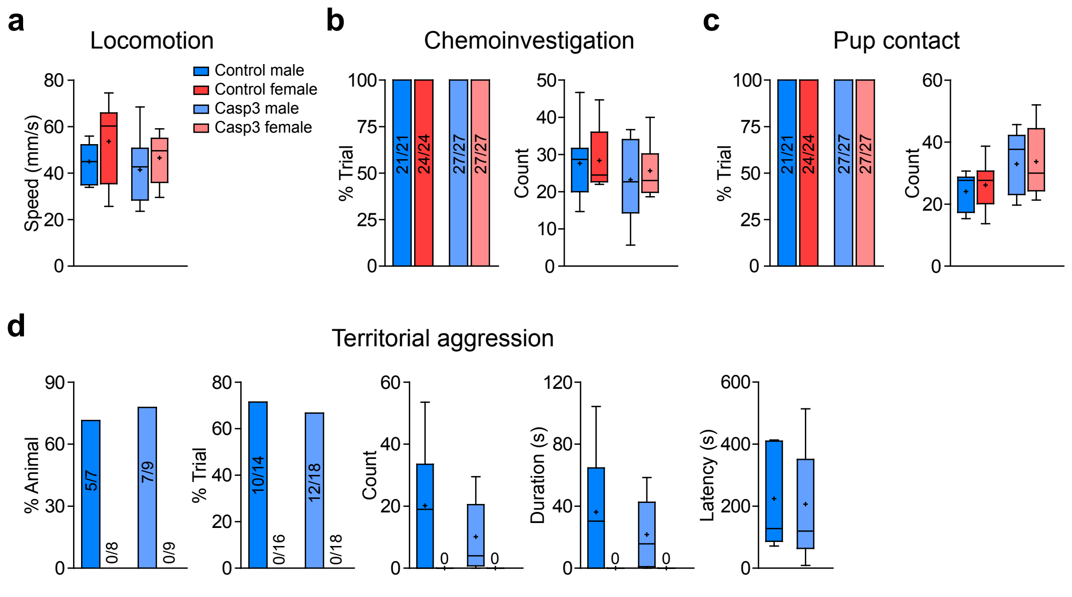

Supplementary Figure 7. Effects of *Esr1+* neuron ablation on other behaviors. Comparison of locomotion (a), chemoinvestigation of a female (b), contacts of pups (c) and territorial aggression behaviors (d) among Esr1^{Cre} animals of both sexes injected bilaterally with Casp3 virus and control animals. No differences were found. N=9 Casp3 and 7 control male and 9 Casp3 and 8 control female. Two-way ANOVA followed by Bonferroni post-hoc tests.

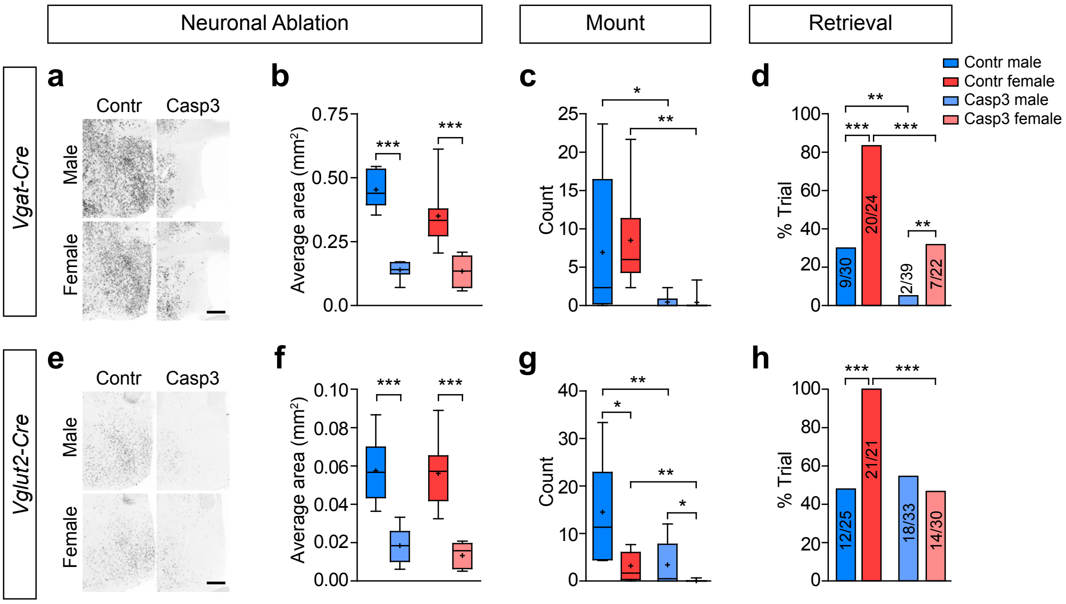

Supplementary Figure 8. Effects of ablating mPOA *Vgat+* and *Vglut2+* neurons on male-typical mating and pup retrieval. a-d. Behavioral effects after ablation of *Vgat+* neurons. a. AAVs encoding Cre-inducible taCasp3 were injected bilaterally into the mPOA of $Vgat^{Cre}$ animals to ablate *Vgat+* neurons. Control animals included Cre mice injected with AAVs encoding mCherry and wildtype littermates injected with the taCasp3 virus. Example images of post-hoc in situ staining of *GAD1* in control (Contr) and experimental (Casp3) animals of both sexes. b. Quantification of neuronal ablation by measuring stained areas of *GAD1+* signals in the mPOA. Two-way ANOVA followed by Bonferroni post-tests showed: virus effect, F(1, 35)=129.52, p<0.0001, sex effect, F(1,35)=5.41, p=0.0259, interaction effect, F(1, 35)=4.42, p=0.0428. c. Ablation of *Vgat+* neuron significantly decreased mounting behavior towards females in both genders. Two-way ANOVA, virus effect, F(1, 35)=16.16, p<0.001, sex effect, F(1,35)=0.18, p=0.6768, interaction effect, F(1, 35)=0.2, p=0.659. d. Ablation of *Vgat+* neurons significantly decreased pup retrieval behavior in both sexes but sex differences in this behavior remained. N=13 Casp3 and 10 control male and 8 Casp3 and 8 control female. Fisher's exact test. **e-h.** Behavioral effects after ablation of *Vglut2+* neurons. e. AAVs encoding Cre-inducible taCasp3 were injected bilaterally into the mPOA of $Vglut2^{Cre}$ animals to ablate *Vglut2+* neurons. Control animals were Cre mice injected with AAVs encoding mCherry. Example images of Post-hoc in situ staining of *Vglut2* in control and experimental animals of both sexes. f. Quantification of neuronal ablation by measuring *Vglut2+* signals in the mPOA. Two-way ANOVA followed by Bonferroni post-tests showed: virus effect, F(1, 34)=95.50, p<0.0001, sex effect, F(1,34)=0.62, p=0.4367, interaction effect, F(1, 34)=0.2, p=0.6568. g. Ablation of *Vglut2+* neurons significantly decreased mounting behavior in both sexes but the sex difference still existed. Two-way ANOVA, virus effect, F(1, 34)=14.44, p<0.001, sex effect, F(1,34)=15.25, p<0.001, interaction effect, F(1, 34)=4.55, p<0.05. h. Ablation of *Vglut2+* neurons abolished sex differences in pup retrieval behavior by specifically reducing this behavior in females. Fisher's exact test. N=12 Casp3 and 9 control male and 10 Casp3 and 7 control female. Scale bar, 300μm. * p< 0.05, ** p< 0.01, *** p < 0.001.

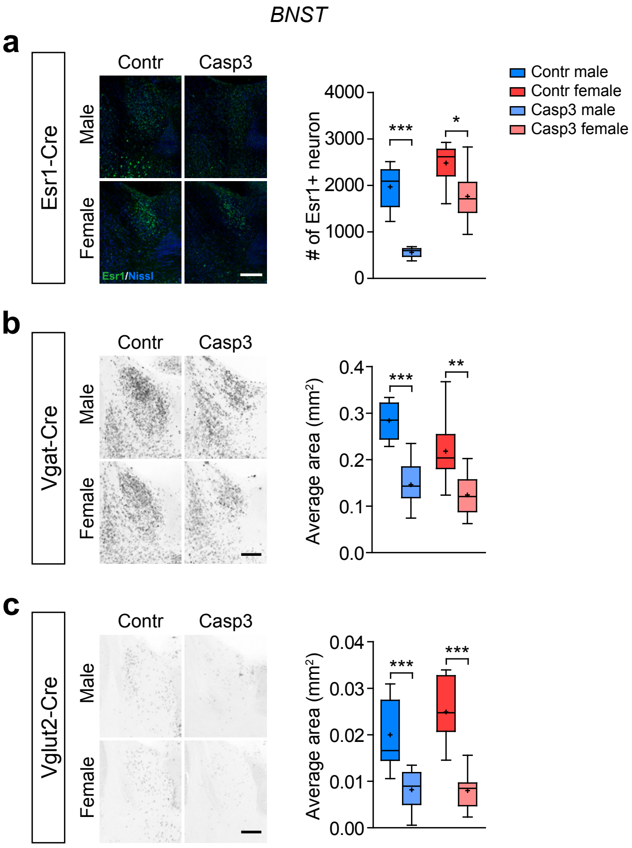

Supplementary Figure 9. taCasp3 induced neuronal ablation in nearby brain regions. Quantification and comparison of Esr1 immunostaining (a), *GAD1* (b) and *Vglut2* mRNA (c) in bed nucleus of the stria terminalis (BNST) in Esr1^{Cre}, Vgat^{Cre}, or Vglut2^{Cre} animals injected with taCasp3 AAVs or control animals. Representative images shown on the left and quantifications on the right. Scale bar, 200µm. Two-way ANOVA followed by Bonferroni post-tests were carried out. For Esr1^{Cre} animals, virus effect, $F(1, 22)=38.8$, $p<0.0001$, sex effect, $F(1,22)=25.16$, $p<0.0001$, interaction effect, $F(1, 22)=4.09$, $p=0.0554$; for Vgat^{Cre} animals, virus effect, $F(1, 35)=49.88$, $p<0.0001$, sex effect, $F(1,35)=7.14$, $p=0.0113$, interaction effect, $F(1, 35)=1.77$, $p=0.1919$; for Vglut2^{Cre} animals, virus effect, $F(1, 34)=63.6$, $p<0.0001$, sex effect, $F(1,34)=1.71$, $p=0.1992$, interaction effect, $F(1, 34)=2.01$, $p=0.1655$. Esr1^{Cre} : N=8 Casp3 and 5 control male and 6 Casp3 and 7 control female. Vgat^{Cre} : N=13 Casp3 and 10 control male and 8 Casp3 and 8 control female. Vglut2^{Cre} : N=12 Casp3 and 9 control male and 10 Casp3 and 7 control female. * $p< 0.05$, ** $p< 0.01$, *** $p < 0.001$.

Supplementary Table 1. Optogenetically induced behaviors towards single or compound stimuli

Gender	Mouse ID	Stimulus	12mW 40Hz			5mW 20Hz		
			Mount	Retrieval	Mount and retrieval	Mount	Retrieval	Mount and retrieval
Female	fm15	single	0.13	1.00		0.00	0.63	
		compound	0.63	0.25	0.13	0.13	0.50	0.25
	fm16	single	0.43	0.70		0.00	0.00	
		compound	0.00	0.25	0.00	0.00	0.00	0.00
	fm17	single	0.80	0.70		0.75	0.75	
		compound	0.25	0.25	0.25	0.38	0.38	0.25
	fm18	single	0.90	0.80		0.00	0.75	
		compound	0.00	0.88	0.00	0.00	0.50	0.00
	fm20	single	0.60	0.90		0.17	0.33	
		compound	0.38	0.13	0.00	0.29	0.00	0.00
	fm4	single	0.50	0.67				
		compound	0.50	0.30	0.00			
	fm5	single	0.92	0.86				
		compound	0.50	0.30	0.00			
	fm6	single	0.44	1.00				
		compound	0.30	0.20	0.00			
	fm9	single	0.31	0.64				
		compound	0.50	0.00	0.00			
Male	mm102	single	0.29	0.71				
		compound	0.09	0.09	0.09	0.08	0.08	0.00
	mm103	single	0.88	0.63				
		compound	0.10	0.50	0.10	0.00	0.75	0.00
	mm104	single	0.25	0.63				
		compound	0.09	0.09	0.00	0.00	0.17	0.00
	mm105	single	0.25	0.75				
		compound	0.14	0.86	0.00	0.00	0.83	0.00
	mm78	single	0.20	1.00		0.75	0.11	
		compound	0.36	0.00	0.27			
	mm79	single	0.56	0.25				
		compound	0.10	0.10	0.00			
	mm80	single	0.60	1.00		0.86	0.43	
		compound	0.58	0.08	0.33			
	mm91	single	0.86	1.00				
		compound	0.27	0.27	0.18	0.00	0.50	0.00
	mm94	single	1.00	0.86				
		compound	0.18	0.27	0.00	0.00	0.42	0.00
	mm95	single	0.75	0.67				
		compound	0.13	0.13	0.00	0.00	0.33	0.00

1. "single", only an ovariectomized female or scattered pups were introduced during the test; "compound", both a female and pups were introduced.
2. Fraction of light stimulation trials (6-12 in total) that a given behavior occurred were presented.
