# Supplementary material for: Medial preoptic area in mice is capable of mediating sexually dimorphic behaviors regardless of gender
Source: Nat Commun. 2018 Jan 18;9:279. doi: 10.1038/s41467-017-02648-0 (PMC5773506; doi:10.1038/s41467-017-02648-0)
Supplement: Supplementary file 2 — Description of Additional Supplementary Files [file 41467_2017_2648_MOESM2_ESM.pdf]

File Name: Supplementary Movie 1
Description: Optogenetic stimulation in male ChR2 mice evoked mounting behavior towards a hormonal primed ovariectomized female. Blue bars in right corner indicate the period of photostimulation

File Name: Supplementary Movie 2
Description: Optogenetic induced mount in male ChR2 mice occasionally transitioned into rhythmic pelvic thrust.

File Name: Supplementary Movie 3
Description: Optogenetic stimulation in female ChR2 mice evoked mounting behavior towards a female.

File Name: Supplementary Movie 4
Description: Optogenetic induced mounting in female ChR2 mice occasionally transitioned into rhythmic pelvic thrust.

File Name: Supplementary Movie 5
Description: Optogenetic stimulation in C57BL/6 male ChR2 mice elicited mounting behavior towards an intact male mouse.

File Name: Supplementary Movie 6
Description: Optogenetic stimulation in C57BL/6 male ChR2 mice elicited mounting behavior towards a P14 young rat.

File Name: Supplementary Movie 7
Description: Optogenetic stimulation in C57BL/6 male mice failed to elicit mounting behavior towards a fake mouse toy.

File Name: Supplementary Movie 8
Description: Optogenetic stimulation of ChR2 male mice induced time-locked pup retrieval behavior and resulted in a pup being brought back to the nest.

File Name: Supplementary Movie 9
Description: Optogenetic stimulation of ChR2 female mice induced time-locked pup retrieval behavior and resulted in grouping of pups in the absence of a nest.

File Name: Supplementary Movie 10
Description: Optogenetic stimulation of ChR2 female mice induced time-locked retrieval behavior towards fake pups and tended to result in grouping of fake pups. Arrows point to fake pups.

File Name: Supplementary Movie 11

Description: When both a female and scattered pups were given as the stimuli, optogenetically induced mounting and pup retrieval occasionally happened in tandem within a single light stimulation trial.
